# Supplementary material for: Insights into the conservation and diversification of the molecular functions of YTHDF proteins
Source: PLoS Genet. 2023 Oct 10;19(10):e1010980. doi: 10.1371/journal.pgen.1010980 (PMC10617740; doi:10.1371/journal.pgen.1010980)
Supplement: S1 Fig — Same phylogenetic analysis of Fig 1, but with rectangular layout, and indicating the protein and species name of all YTHDFs, and aLRT values (%) for all nodes. The representative proteins labeled in Fig 1B are highlighted with larger typography on the right side of this figure as a reference. (PDF) [file pgen.1010980.s001.pdf]

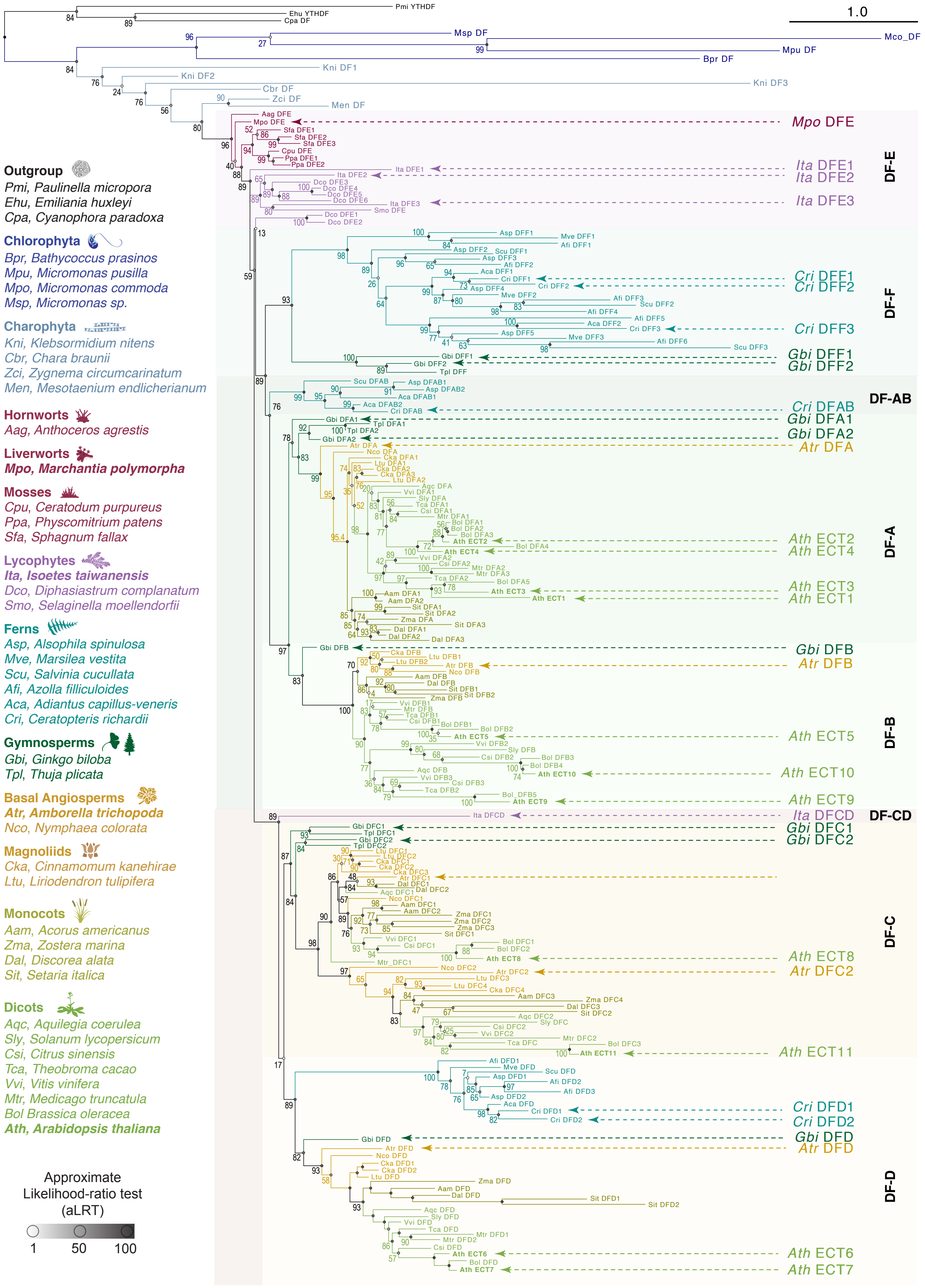


**S1 Fig. Fully annotated phylogenetic tree.** Same phylogenetic analysis of Fig 1, but with rectangular layout, and indicating the protein and species name of all YTHDFs, and aLRT values (%) for all nodes. The representative proteins labeled in Fig 1B are highlighted with larger typography on the right side of this figure as a reference.
