# Supplementary material for: Insights into the conservation and diversification of the molecular functions of YTHDF proteins
Source: PLoS Genet. 2023 Oct 10;19(10):e1010980. doi: 10.1371/journal.pgen.1010980 (PMC10617740; doi:10.1371/journal.pgen.1010980)
Supplement: S2 Fig — Number of genes encoding YTHDF and YTHDC proteins in several species of Archaeoplastida (see Figs 1 and S1 for detailed data on Embryophyta), and closely related taxa. NI, Not Identified. Phylogenetic relationships between groups according to [55,62,100,101] are indicated. Among chlorophytes, YTHDF proteins were found only in species of the Micromonas and Bathycoccus genera of the basal group Mamiellophyceae, although losses in other genera within this group have also occurred (e.g. Ostreococcus [68]). Regarding charophytes, the only sequenced species of their earliest-branching group, Mesostigma viride, has lost YTHDF proteins altogether, but YTHDF-encoding genes are found in four species of later-diverging groups: Klebsormidium nitens, Chara braunii, Zygnema circumcarinatu, and Mesotaenium endlicherianum. (PDF) [file pgen.1010980.s002.pdf]

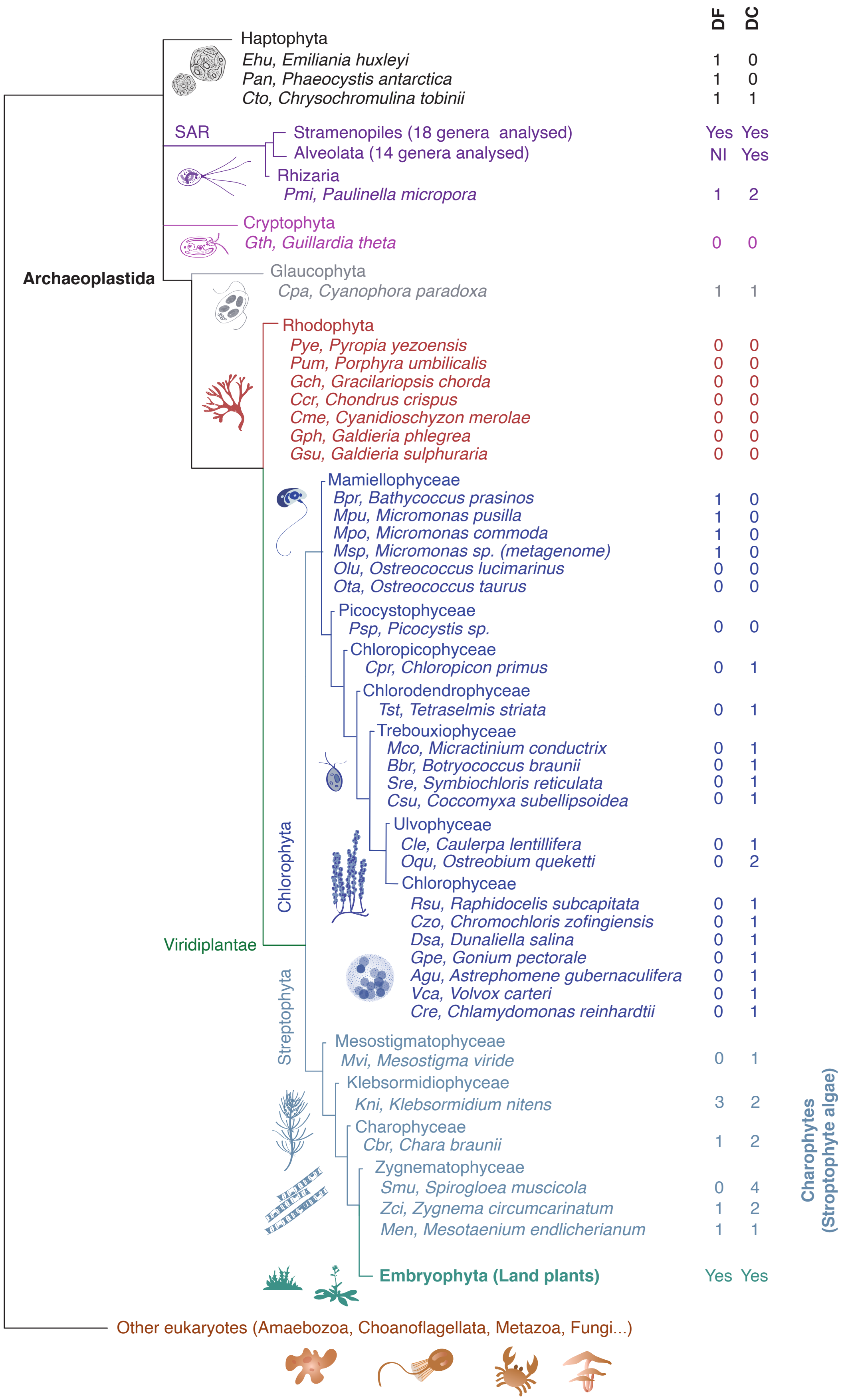

S2 Fig. YTH domain proteins in Archaeoplastida. Number of genes encoding YTHDF and YTHDC proteins in several species of Archaeoplastida (see Figs 1 and S1 for detailed data on Embryophyta), and closely related taxa. NI, Not Identified. Phylogenetic relationships between groups according to [55, 97-99] are indicated. Among chlorophytes, YTHDF proteins were found only in species of the *Micromonas* and *Bathycoccus* genera of the basal group *Mamiellophyceae*, although losses in other genera within this group have also occurred (e.g. *Ostreococcus* [67]). Regarding charophytes, the only sequenced species of their earliest-branching group, *Mesostigma viride*, has lost YTHDF proteins altogether, but YTHDF-encoding genes are found in four species of later-diverging groups: *Klebsormidium nitens*, *Chara braunii, Zygnema circumcarinatu*, and *Mesotaenium endlicherianum*.
