# Supplementary material for: Insights into the conservation and diversification of the molecular functions of YTHDF proteins
Source: PLoS Genet. 2023 Oct 10;19(10):e1010980. doi: 10.1371/journal.pgen.1010980 (PMC10617740; doi:10.1371/journal.pgen.1010980)
Supplement: S3 Fig — Amino acid sequence alignment of the YTH domains of DF proteins in the Viridiplantae species used for the phylogenetic analysis in Fig 1B, highlighting the family members found in green algae (see species abbreviations in S2 Fig). The frequent amino acid substitutions in structural elements of the green algal orthologs are revealed by the absence of the blue shading that indicates percentage identity (Jalview [114]). In some chlorophyte DFs, these substitutions affect the m6A-recognition aromatic cage (red arrows and red/yellow shading), because the two embryophyte-invariant tryptophan residues in the recognition loop (Fig 5A) exhibit changes to proline, histidine or phenylalanine. Chlorophyte DF proteins also contain insertions in their YTH domains, here marked with magenta lines and numbers above the sequences that correspond to collapsed gaps in S4 Fig. The insertions have variable lengths, reaching up to 77 amino acids, and can disrupt the m6A-recognition loop, e.g. the poly-Glycine stretch of Micromonas commoda DF (insertion 10, GGGGGGGGGGGGGGGPR). In the charophyte DF orthologs, the YTH domains have intact aromatic cages and overall higher conservation than those of chlorophytes, but they also differ considerably from the land plant relatives. For example, the sequences of the β1 and α1 structural elements (Fig 5A) have degenerated in one of the three Klebsormidium nitens DF proteins (Kni DF3), and a 156-amino acid insertion protruding from the plant-specific α2-extension is apparent in the only DF protein from Mesotaenium endlicherianum (Men DF), a species that belongs to the sister clade of all embryophytes (S2 Fig). (PDF) [file pgen.1010980.s003.pdf]

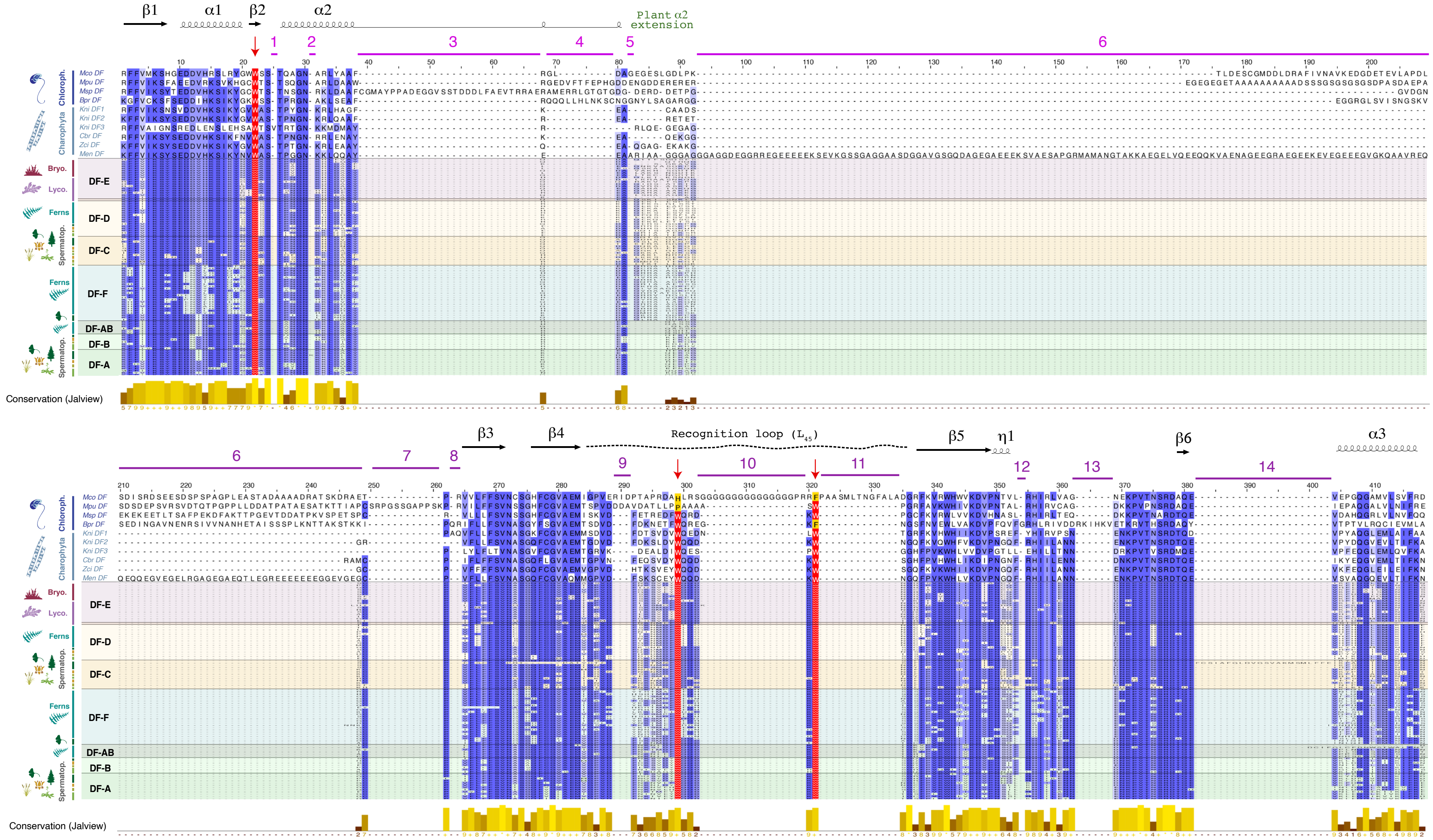

S3 Fig. Conservation of YTHDF proteins in green algae. Amino acid sequence alignment of the YTHDF proteins of the Viridiplantae species used for the phylogenetic analysis in Fig 1B, highlighting the family members found in green algae (see species abbreviations in S2 Fig). The frequent amino acid substitutions in structural elements of the green algal orthologs are revealed by the absence of the blue shading that indicates percentage identity (Jalview [112]). In some chlorophyte DFs, these substitutions affect the m^6A-recognition aromatic cage (red arrows and red/yellow shading), because the two embryophyte-invariant tryptophan residues in the recognition loop (Fig 5A) exhibit changes to proline, histidine or phenylalanine. Chlorophyte DF proteins also contain insertions in their YTH domains, here marked with magenta lines and numbers above the sequences that correspond to collapsed gaps in S4 Fig. The insertions have variable lengths, reaching up to 77 amino acids, and can disrupt the m^6A-recognition loop, e.g. the poly-Glycine stretch of *Micromonas commoda* DF (insertion 10, GGGGGGGGGGGGGGGPR). In the charophyte DF orthologs, the YTH domains have intact aromatic cages and overall higher conservation than those of chlorophytes, but they also differ considerably from the land plant relatives. For example, the sequences of the β1 and α1 structural elements (Fig 5A) have degenerated in one of the three *Klebsormidium nitens* DF proteins (*Kni* DF3), and a 156-amino acid insertion protruding from the plant-specific α2-extension is apparent in the only DF protein from *Mesotaenium endlicherianum* (*Men* DF), a species that belongs to the sister clade of all embryophytes (S2 Fig).
