# Supplementary material for: Insights into the conservation and diversification of the molecular functions of YTHDF proteins
Source: PLoS Genet. 2023 Oct 10;19(10):e1010980. doi: 10.1371/journal.pgen.1010980 (PMC10617740; doi:10.1371/journal.pgen.1010980)
Supplement: S4 Fig — Amino acid sequence alignment of the YTH domains of DF proteins in plant taxa that diverged before the evolution of flowers. A few angiosperm YTHDFs are included in the analysis for comparison. Magenta-colored numbers at the top indicate the positions of amino acid insertions in one or few proteins. Because these insertions create gaps in the aligned sequences of the other homologs, they have been hidden to gain space and clarity, but the corresponding sequences can be found in S3 Fig and S5 Dataset. Red arrows under the alignment point to the conserved aromatic residues that contact m6A, and a green arrow marks the Asp-to-Asn substitution, present in YTHDCs, that increases the affinity for m6A by 15-fold [61]. This substitution is also found in the plant DF-B clade [26], and here revealed in fern DF-F proteins as well. Blue-white coloring and the conservation and consensus tracks at the bottom are computed by Jalview [114] as in S3 Fig. (PDF) [file pgen.1010980.s004.pdf]

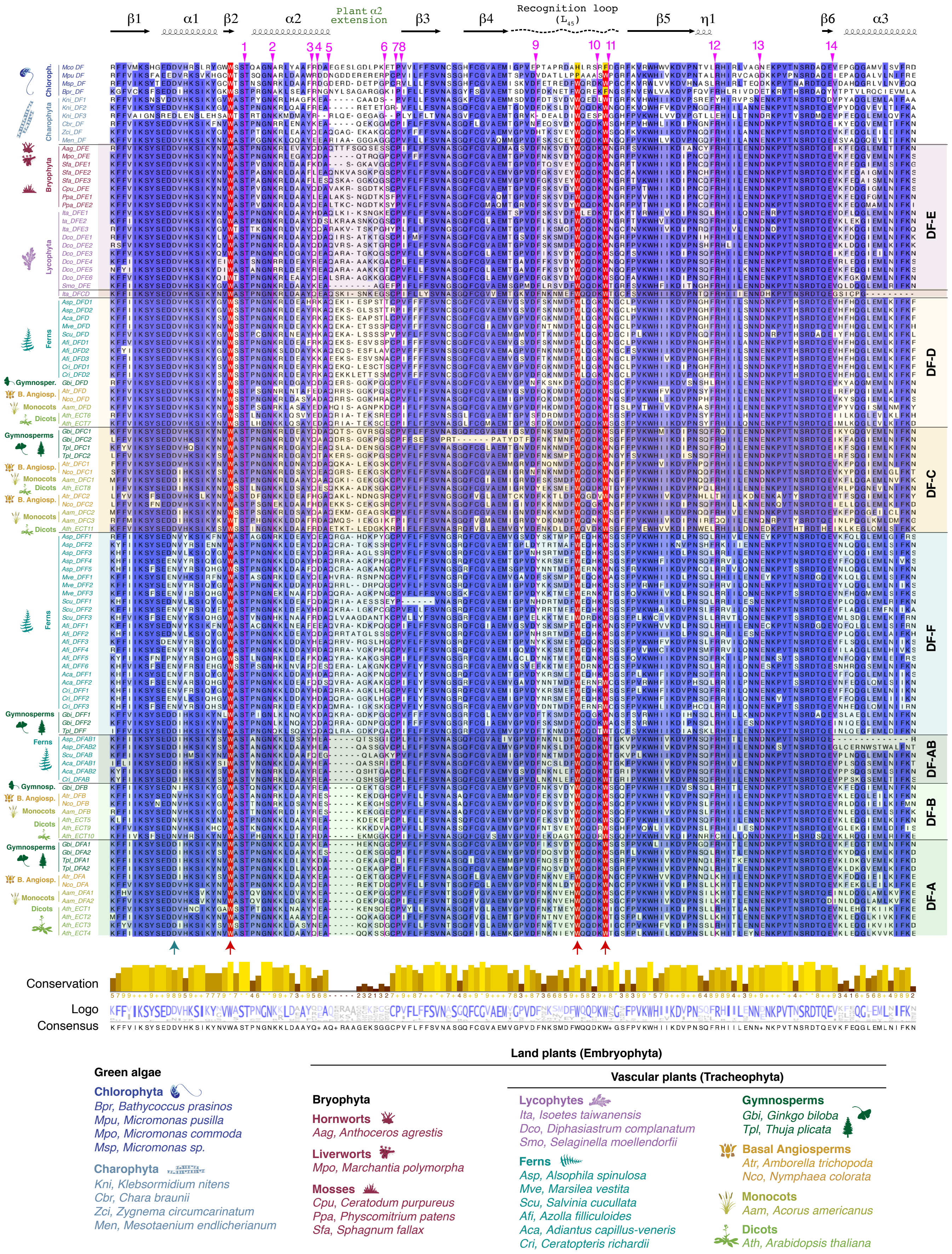

S4 Fig. Conservation of YTHDF proteins in cryptogams and gymnosperms. Amino acid sequence alignment of the YTH domains of DF proteins in plant taxa that diverged before the evolution of flowers. A few angiosperm YTHDFs are included in the analysis for comparison. Magenta-colored numbers at the top indicate the positions of amino acid insertions in one or few proteins. Because these insertions create gaps in the aligned sequences of the other homologs, they have been hidden to gain space and clarity, but the corresponding sequences can be found in S3 Fig and S5 Dataset. Red arrows under the alignment point to the conserved aromatic residues that contact m^6A, and a green arrow marks the Asp-to-Asn substitution, present in YTHDCs, that increases the affinity for m^6A by 15-fold [61]. This substitution is also found in the plant DF-B clade [26], and here revealed in fern DF-F proteins as well. Blue-white coloring and the conservation and consensus tracks at the bottom are computed by Jalview [112] as in S3 Fig.
