# Supplementary material for: Insights into the conservation and diversification of the molecular functions of YTHDF proteins
Source: PLoS Genet. 2023 Oct 10;19(10):e1010980. doi: 10.1371/journal.pgen.1010980 (PMC10617740; doi:10.1371/journal.pgen.1010980)
Supplement: S5 Fig — 9-day-old primary transformants expressing ECT1, ECT2 or ECT4 fused to mCherry in the te234 background. All transgenes are expressed from gDNA under the control of the ECT2 promoter. mCherry fluorescence on the right panels show the variability in transgene expression levels with this setup. Additional genotypes in the top panels are shown as a reference. Scale bars are 0.5 mm. (PDF) [file pgen.1010980.s005.pdf]

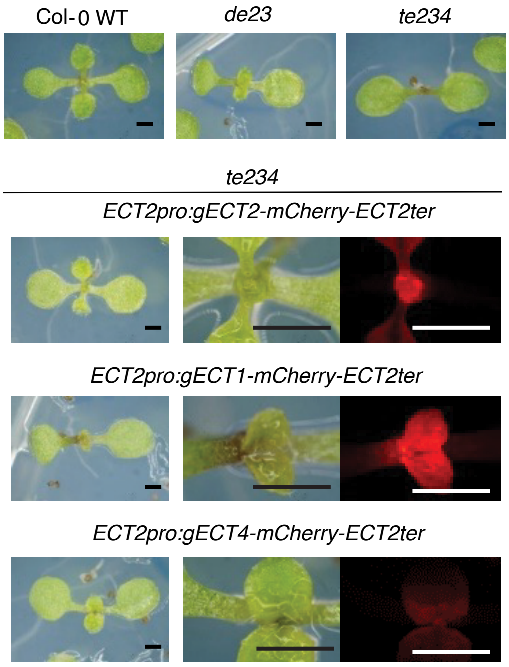

S5 Fig. Expression of gDNA constructs of *ECT1, ECT2* and *ECT4* under the control of the *ECT2* promoter. 9-day-old primary transformants expressing ECT1, ECT2 or ECT4 fused to mCherry in the *te234* background. All transgenes are expressed from gDNA under the control of the *ECT2* promoter. mCherry fluorescence on the right panels show the variability in transgene expression levels with this setup. Additional genotypes in the top panels are shown as a reference. Scale bars are 0.5 mm.
