# Supplementary material for: Insights into the conservation and diversification of the molecular functions of YTHDF proteins
Source: PLoS Genet. 2023 Oct 10;19(10):e1010980. doi: 10.1371/journal.pgen.1010980 (PMC10617740; doi:10.1371/journal.pgen.1010980)
Supplement: S6 Fig — (A) Raw percentages of seedlings with first true leaves >0.5 mm at 10 days after germination among primary transformants of te234 or rdr6-12/te234 plants transformed with US7Yp:cECT2-mCherry-OCSt (cDNA) or US7Yp:mCherry-OCSt (control) compared to ECT2p:gECT2-mCherry-ECT2t (gDNA). (B) Expression pattern in 9-day-old seedlings of the indicated genotypes (T2 for US7Yp-driven constructs, and T5 for ECT2p:gECT2-mCherry-ECT2t). Fluorescence and protein abundance is typically higher in plants expressing free mCherry (US7Yp:mCherry-OCSt) than in fusions of mCherry with ECTs (see S8 and S15 Figs). Although the expression pattern of US7Yp:cECT2-mCherry-OCSt is identical to that of ECT2p:gECT2-mCherry-ECT2t, the fluorescence level and protein abundance observed is typically lower among cDNA lines in the te234 background. Scale bars are 1 mm for images of aerial tissues (upper panels), and 0.1 mm for roots (lower panels). (PDF) [file pgen.1010980.s006.pdf]

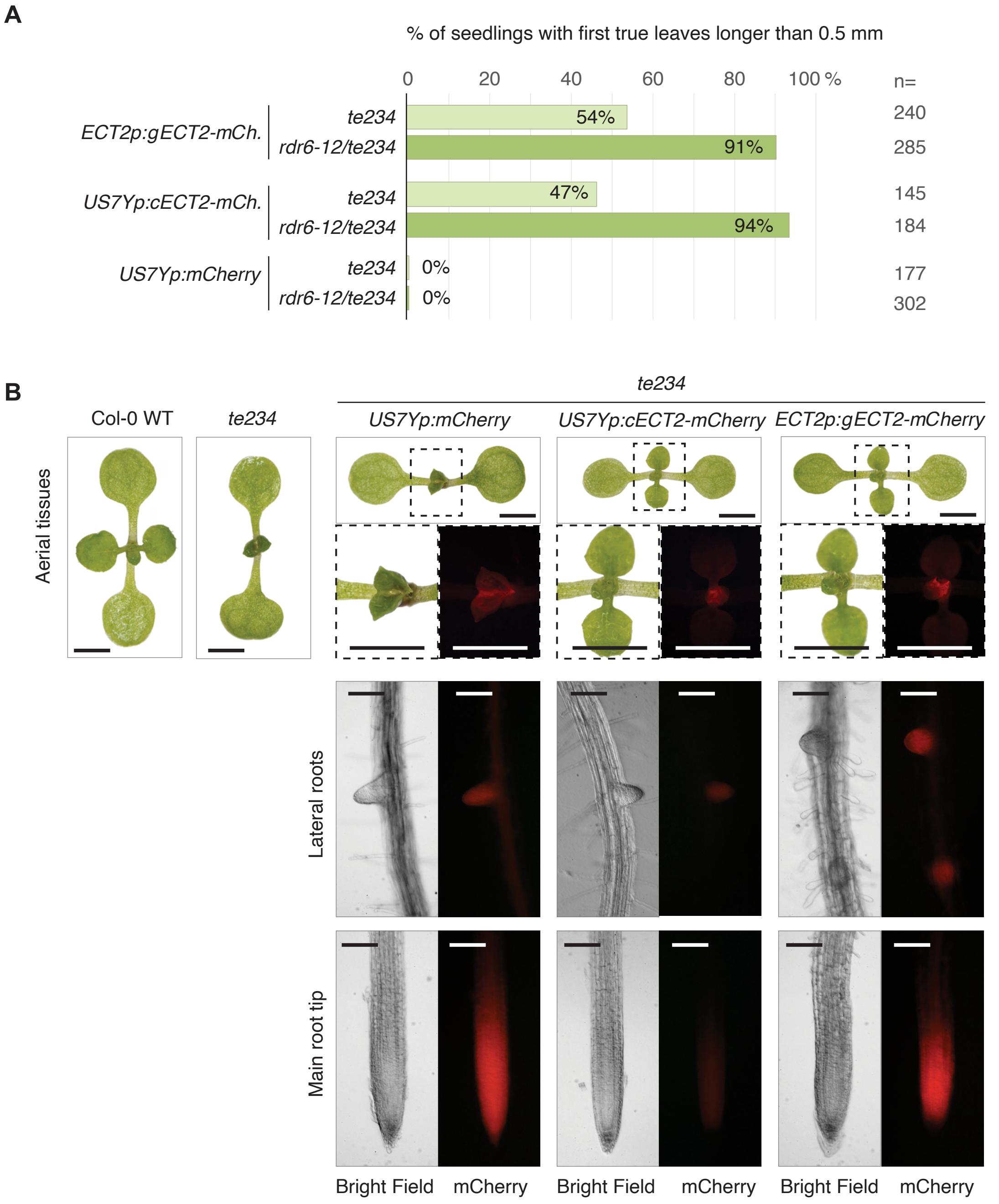

S6 Fig. Expression of *cECT2-mCherry* driven by the ribosomal protein *uS7Y* promoter can rescue late leaf emergence in *ect2-1/ect3-1/ect4-2* (*te234*) seedlings. (A). Raw percentages of seedlings with first true leaves >0.5 mm at 10 days after germination among primary transformants of *te234* or *rdr6-12/te234* plants transformed with *uS7Yp:cECT2-mCherry-OCSt* (cDNA) or *uS7Yp:mCherry-OCSt* (control) compared to *ECT2p:gECT2-mCherry-ECT2t* (gDNA). **(B)** Expression pattern in 9-day-old seedlings of the indicated genotypes (T2 for *US7Yp*-driven constructs, and T5 for *ECT2p:gECT2-mCherry-ECT2t*). Fluorescence and protein abundance is typically higher in plants expressing free mCherry (*US7Yp:mCherry-OCSt*) than in fusions of mCherry with ECTs (see S8 and S15 Figs). Although the expression pattern of *US7Yp:cECT2-mCherry-OCSt* is identical to that of *ECT2p:gECT2-mCherry-ECT2t*, the fluorescence level and protein abundance observed is typically lower among cDNA lines in the *te234* background. Scale bars are 1 mm for images of aerial tissues (upper panels), and 0.1 mm for roots (lower panels).
