# Supplementary material for: Insights into the conservation and diversification of the molecular functions of YTHDF proteins
Source: PLoS Genet. 2023 Oct 10;19(10):e1010980. doi: 10.1371/journal.pgen.1010980 (PMC10617740; doi:10.1371/journal.pgen.1010980)
Supplement: S7 Fig — (A-D) Absolute complementation rates for each independent transformation in te234 (A, B, D) or rdr6-12/te234 (C) plants given by the percentage of primary transformants (T1s) with first true leaves bigger than 0.5 mm at 10 days after germination for US7Yp:cECT(X)-mCherry-OCSt (A, C), US7Yp:cECT(X)IDR/cECT(Y)YTH-mCherry-OCSt chimeras (B, C), or US7Yp:cYTHDF(X)-mCherry-OCSt heterologous constructs (C, D). Numbers over the bars indicate the total number of transformants. Asterisks indicate absence of mCherry fluorescence (expression) among all retrieved transformants, or very faint fluorescence that underwent silencing in the next generation (T2). S6 Dataset contains detailed T1 counts and the calculation of complementation rates. (PDF) [file pgen.1010980.s007.pdf]

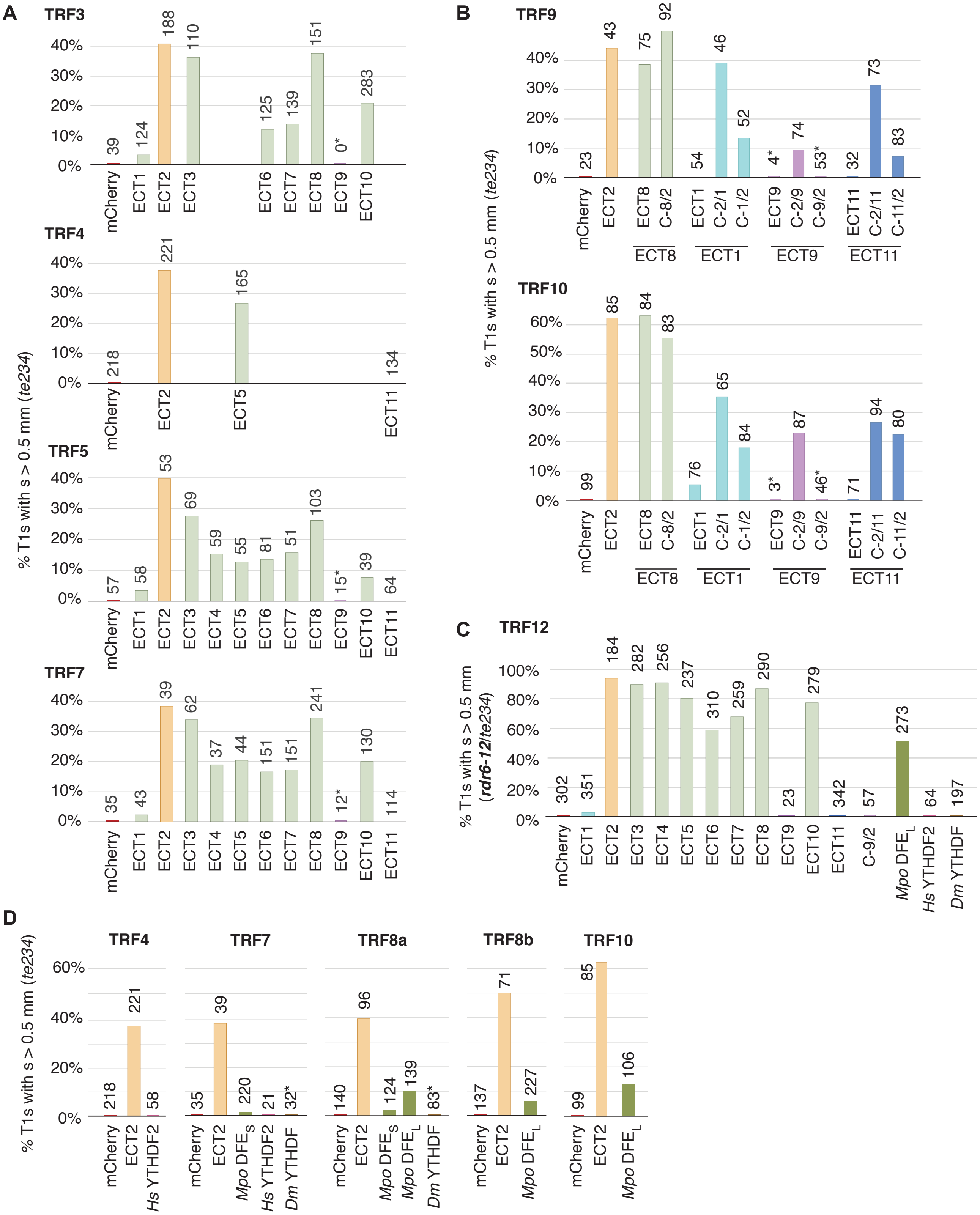

S7 Fig. Complementation rates (raw data). **(A-D)** Absolute complementation rates in each independent transformation in *te234* (A,B,D) or *rdr6-12/te234* (C) plants given by the percentage of primary transformants (T1s) with first true leaves bigger than 0.5 mm at 10 days after germination (DAG) for *uS7Bp:cECT(X)-mCherry-OCSt* (A, C), *uS7Bp:cECT(X)*$_{IDR}$*/cECT(Y)*$_{YTH}$*-mCherry-OCSt* chimeras (B, C), or *uS7Bp:cYTHDF(X)-mCherry-OCSt* heterologous constructs (C,D). Numbers over the bars indicate the total number of transformants. Asterisks indicate absence of mCherry fluorescence (expression) among all retrieved transformants, or very faint fluorescence that underwent silencing in the next generation (T2). S6 Dataset contains detailed T1 counts and the calculation of complementation rates.
