# Supplementary material for: Insights into the conservation and diversification of the molecular functions of YTHDF proteins
Source: PLoS Genet. 2023 Oct 10;19(10):e1010980. doi: 10.1371/journal.pgen.1010980 (PMC10617740; doi:10.1371/journal.pgen.1010980)
Supplement: S8 Fig — (A) Representative 10-day-old te234/US7Yp:cECT(X)-mCherry-OCSt T1 seedlings with their fluorescent signal and genetic background controls. Bars represent weighed averages of complementation over 2–5 transformations (Fig 2C). Scale bars are 1 mm. (B) Levels of protein expression from US7Yp:cECT(X)-mCherry-OCSt constructs in independent lines (L) of 9-day-old T2 seedlings assessed by α-mCherry western blot. All genotypes are in the te234 background. Lines with single insertions and the highest complementation capacity (largest true leaves at 9 days after germination in T2) were selected for the analysis. ECT9 is not included because we could not observe fluorescence in the T2 generation for any of the few lines obtained, due to silencing. te234 ECT2p:gECT2-mCherry-ECT2t lines [16] are included in both membranes as a reference. Lanes marked with X in the lower membrane correspond to either lines irrelevant for this study, or left empty due to defects in the wells. Ponceau-staining is shown as loading control. (PDF) [file pgen.1010980.s008.pdf]

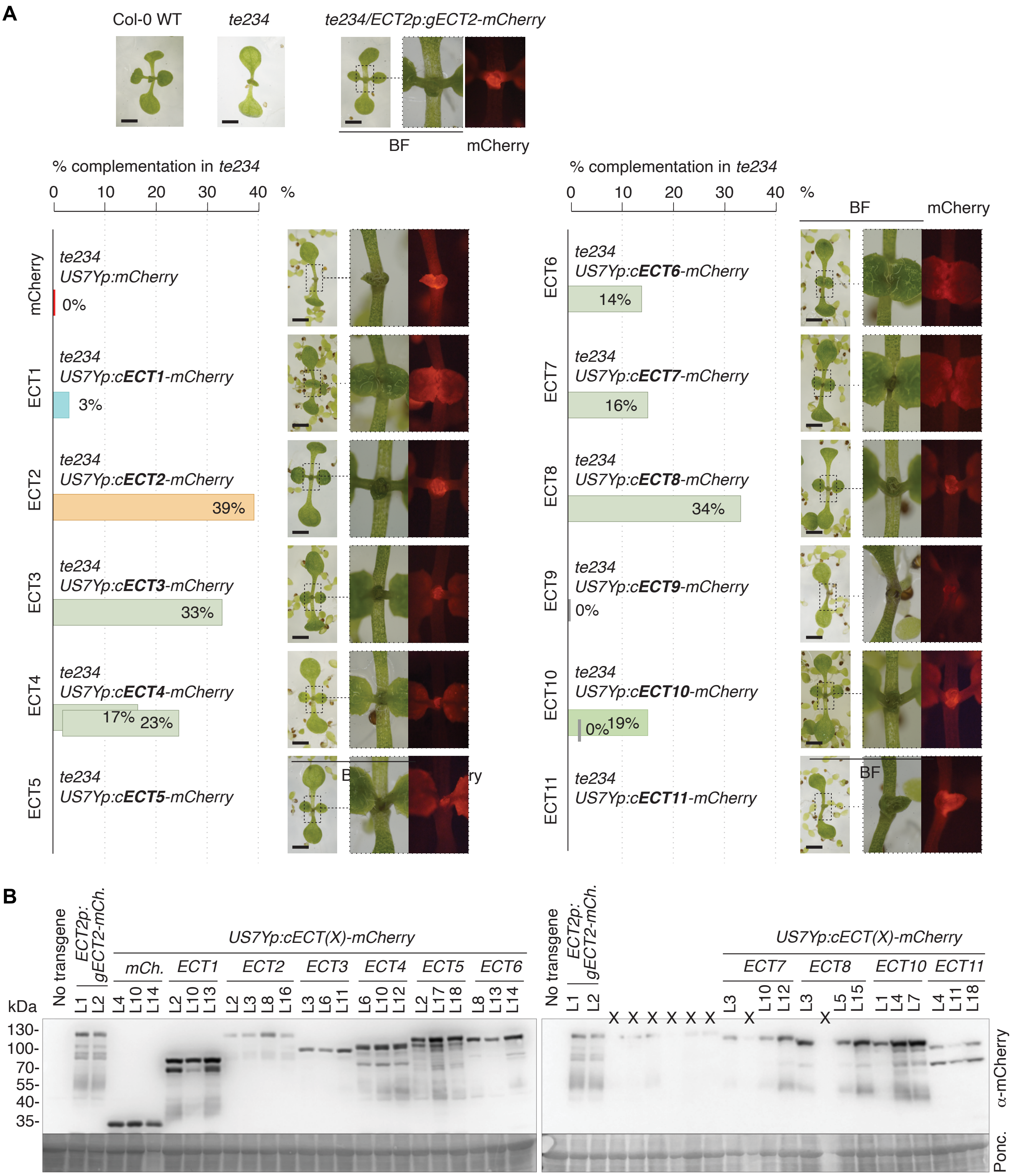

S8 Fig. Expression of *US7Yp:cECT(X)-mCherry-OCSt* constructs in *te234* plants. (A) Representative 10-day-old *te234/US7Yp:cECT(X)-mCherry-OCSt* T1 seedlings with their fluorescent signal and genetic background controls. Bars represent weighed averages of complementation over 2-5 transformations (Fig 2C). Scale bars are 1 mm. **(B)** Levels of protein expression from *US7Yp:cECT(X)-mCherry-OCSt* constructs in independent lines (L) of 9-day-old T2 seedlings assessed by α-mCherry western blot. All genotypes are in the *te234* background. Lines with single insertions and the highest complementation capacity (largest true leaves at 9 days after germination in T2) were selected for the analysis. ECT9 is not included because we could not observe fluorescence in the T2 generation for any of the few lines obtained, due to silencing. *te234 ECT2p:gECT2-mCherry-ECT2t* lines [16] are included in both membranes as a reference. Lanes marked with X in the lower membrane correspond to either lines irrelevant for this study, or left empty due to defects in the wells. Ponceau-staining is shown as loading control.
