# Supplementary material for: Insights into the conservation and diversification of the molecular functions of YTHDF proteins
Source: PLoS Genet. 2023 Oct 10;19(10):e1010980. doi: 10.1371/journal.pgen.1010980 (PMC10617740; doi:10.1371/journal.pgen.1010980)
Supplement: S9 Fig — Estimated transformation efficiency (average of 6 plates) of each construct for all the independent transformation batches of te234 (TRF1-10) or rdr6-12/te234 (TRF12) plants used in this study. TRF8 was screened twice (TRF8a and TRF8b). Red-to-blue colouring in the te234 background reflects the variability in transformation efficiencies across constructs and batches, to highlight the consistently low recovery of transformants for Arabidopsis thaliana (Ath) ECT9 and Homo sapiens (Hs) YTHDF2. (PDF) [file pgen.1010980.s009.pdf]

| | *te234* | | | | | | | | | *rdr6-12 te234* |
|---|---|---|---|---|---|---|---|---|---|---|
| **T1s per plate (~2000 seeds)** | **TRF1** | **TRF3** | **TRF4** | **TRF5** | **TRF7** | **TRF8a** | **TRF8b** | **TRF9** | **TRF10** | **TRF12** |
| **mCherry** | 35.0 | 6.5 | 36.3 | 9.5 | 5.8 | 23.3 | 22.8 | 3.8 | 16.5 | 50.3 |
| **ECT1** | | 20.7 | | 9.7 | 7.2 | | | 9.0 | 12.7 | 58.5 |
| **ECT2** | 37.0 | 31.3 | 36.8 | 8.8 | 6.5 | 16.0 | 11.8 | 7.2 | 14.2 | 30.7 |
| **ECT3** | | 18.3 | | 11.5 | 10.3 | | | | | 56.4 |
| **ECT4** | | | | 9.8 | 6.2 | | | | | 42.7 |
| **ECT5** | | | 27.5 | 9.2 | 7.3 | | | | | 39.5 |
| **ECT6** | | 20.8 | | 13.5 | 25.2 | | | | | 51.7 |
| **ECT7** | | 23.2 | | 8.5 | 25.2 | | | | | 51.8 |
| **ECT8** | | 25.2 | | 17.2 | 40.2 | | | 12.5 | 14.0 | 48.3 |
| **ECT9** | | 0.0 | | 2.5 | 2.0 | | | 0.7 | 0.5 | 4.6 |
| **ECT10** | | 47.2 | | 6.5 | 21.7 | | | | | 69.8 |
| **ECT11** | | | 22.3 | 10.7 | 19.0 | | | 5.3 | 11.8 | 57.0 |
| **C-1/2** | | | | | | | | 8.7 | 14.0 | |
| **C-2/1** | | | | | | | | 7.7 | 10.8 | |
| **C-9/2** | | | | | | | | 8.8 | 7.7 | 20.5 |
| **C-2/9** | | | | | | | | 12.3 | 14.5 | |
| **C-11/2** | | | | | | | | 13.8 | 13.3 | |
| **C-2/11** | | | | | | | | 12.2 | 15.7 | |
| **C-8/2** | | | | | | | | 15.3 | 13.8 | |
| ***Mpo* DFE$_S$** | | | | | 36.7 | 20.7 | | | | |
| ***Mpo* DFE$_L$** | | | | | | 23.2 | 18.9 | | | 45.5 |
| ***Hs* YTHDF2** | | | 9.7 | | 3.5 | | | | | 10.7 |
| ***Dm* YTHDF** | | | | | 5.3 | 13.8 | | | | 32.8 |

Transformation efficiency (average) *te234:* 15.3 T1s / plate
*rdr6-12/te234:* 41.9 T1s / plate

**S9 Fig. Transformation Efficiency.** Estimated transformation efficienty (average of 6 plates) of each construct for all the independent transformation batches of *te234* (TRF1-10) or *rdr6-12/te234* (TRF12) plants used in this study. TRF8 was screened twice (TRF8a and TRF8b). Red-to-blue colouring in the *te234* background reflect the variability in transformation efficiencies across constructs and batches, to highlight the consistently low recovery of transformants for *Ath* ECT9 and *Hs* YTHDF2.
