# Supplementary material for: Insights into the conservation and diversification of the molecular functions of YTHDF proteins
Source: PLoS Genet. 2023 Oct 10;19(10):e1010980. doi: 10.1371/journal.pgen.1010980 (PMC10617740; doi:10.1371/journal.pgen.1010980)
Supplement: S10 Fig — (A) Developmental stages of plants of the indicated genotypes. DAG, days after germination. (B) Same as in A for primary transformants (T1s) of the indicating transgenes, all expressed from the US7Y promoter with mCherry fused at the C-terminus. Dashed outlines at 10 DAG are magnified below each panel to show mCherry fluorescence. Several different independent lines of the same age are shown for the genotypes exhibiting the strongest developmental defects. Plants in A and B were grown in parallel. (PDF) [file pgen.1010980.s010.pdf]

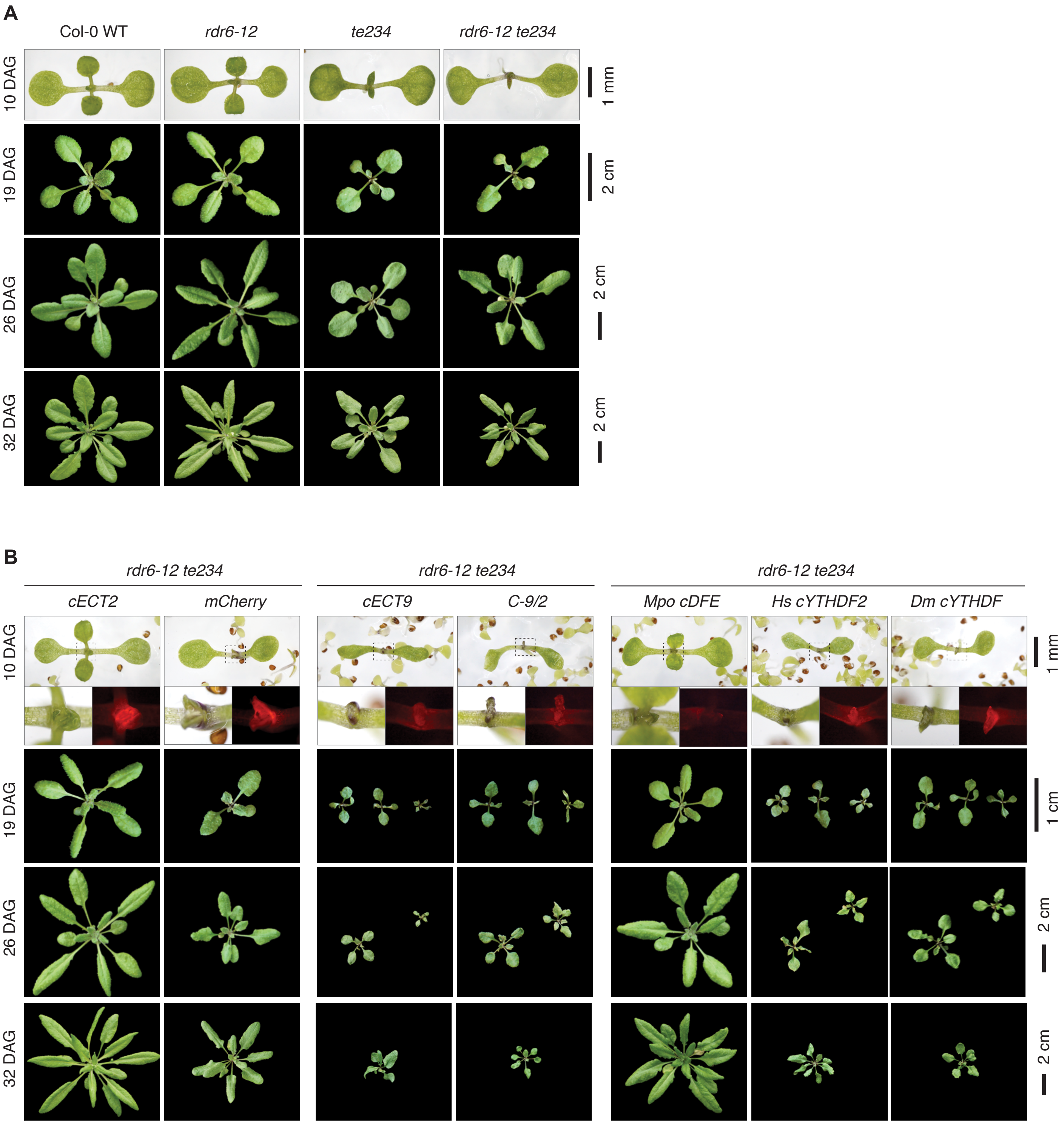

S10 Fig. Phenotypic characterization of selected transgenic lines in *rdr6-12/te234* and relevant genetic backgrounds. (A) Developmental stages of plants of the indicated genotypes. DAG, days after germination. **(B)** Same as in A for primary transformants (T1s) of the indicating transgenes, all expressed from the *US7Y* promoter with mCherry fused at the C-terminus. Dashed outlines at 10 DAG are magnified below each panel to show mCherry fluorescence. Several different independent lines of the same age are shown for the genotypes exhibiting the strongest developmental defects. Plants in A and B were grown in parallel.
