# Supplementary material for: Insights into the conservation and diversification of the molecular functions of YTHDF proteins
Source: PLoS Genet. 2023 Oct 10;19(10):e1010980. doi: 10.1371/journal.pgen.1010980 (PMC10617740; doi:10.1371/journal.pgen.1010980)
Supplement: S11 Fig — Rosettes of 24-day-old primary transformants expressing the indicated transgenes in the rdr6-12 background, grown in parallel with rdr6-12 and rdr6-12/te234 controls. (PDF) [file pgen.1010980.s011.pdf]

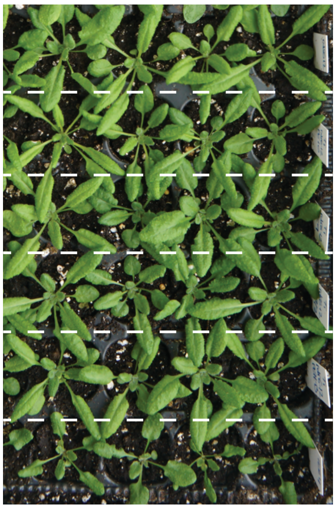


**S11 Fig. Expression of *US7Yp:cECT9-mCherry-OCSt* in the *rdr6-12* background has no obvious phenotypic effect.** Rosettes of 24-day-old primary transformants expressing the indicated transgenes in the *rdr6-12* background, grown in parallel with *rdr6-12* and *rdr6-12/te234* controls.
