# Supplementary material for: Insights into the conservation and diversification of the molecular functions of YTHDF proteins
Source: PLoS Genet. 2023 Oct 10;19(10):e1010980. doi: 10.1371/journal.pgen.1010980 (PMC10617740; doi:10.1371/journal.pgen.1010980)
Supplement: S12 Fig — Expression levels of ECT1-ECT11 across different tissues according to public mRNA-Seq data [73]. TPM, transcripts per million. (PDF) [file pgen.1010980.s012.pdf]

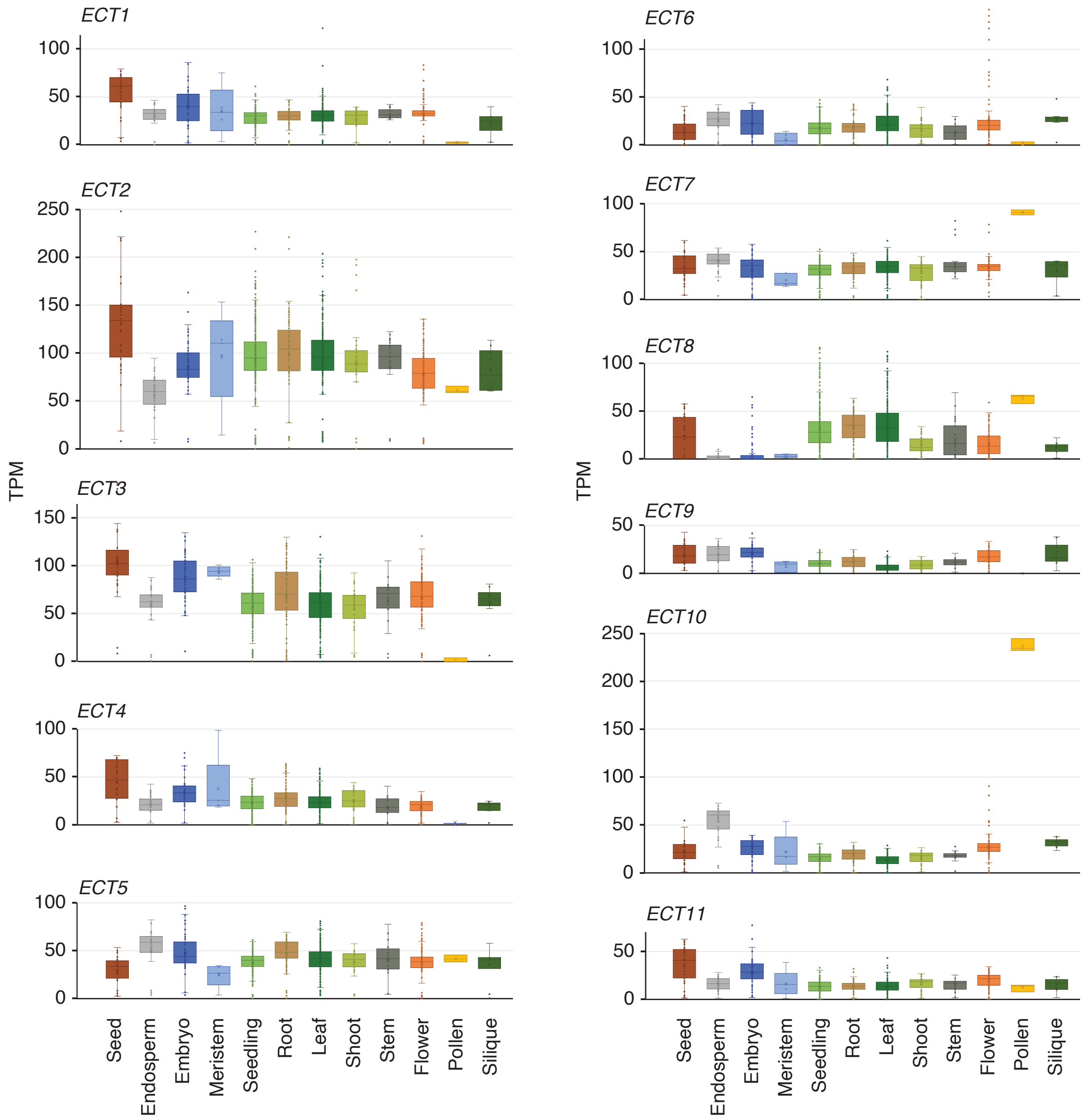

S12 Fig. Expression levels of *ECT* paralogs in tissues of *Arabidopsis thaliana*. Expression levels of *ECT1-ECT11* across different tissues according to public mRNA-Seq data [72]. TPM, transcripts per million.
