# Supplementary material for: Insights into the conservation and diversification of the molecular functions of YTHDF proteins
Source: PLoS Genet. 2023 Oct 10;19(10):e1010980. doi: 10.1371/journal.pgen.1010980 (PMC10617740; doi:10.1371/journal.pgen.1010980)
Supplement: S13 Fig — (A) Western blot using antibodies against GFP (that recognize TFP) in different ECT1p:gECT1-TFP-ECT1t independent lines. Ponceau (Ponc.) staining of the membrane is used as loading control. (B) Northern blot using the probe (P) specified in Fig 3G to detect ECT1 mRNA. Although the probe recognizes specifically ECT1 in the ECT1p:gECT1-TFP-ECT1t Line #21 (marked with an asterisk in A), the endogenous expression levels of ECT1 in Col-0 wild type are below detection limit by northern blot. (C) Schematic representation of the Arabidopsis thaliana ECT1 locus (At3g03950). Exons are represented as boxes and introns as lines. Untranslated regions (UTRs) are coloured grey, the sequence encoding the YTH domain is purple, and the rest of the ECT1-coding sequence is black. The IDs and positions of the T-DNA insertions assigned to ect1-1, ect1-2 and ect1-3 alleles are marked, and so is the location of primers used for their genotyping. (D-F) 1% agarose gels showing EtBr-stained PCR fragments corresponding to the genotyping of ect1-1 (D), ect1-2 (E) and ect1-3 (F) in plants germinated from seeds provided by the Nottingham Arabidopsis Stock Center (NASC) as indicated. The primer set for each PCR (‘T-DNA’ detects the insertion, and ‘WT’ detects the wild type allele) and the length of the resulting amplicons are indicated to the left of each gel. The sequence of all primers can be found in S2 Table. The progeny of plants homozygous for each T-DNA insertion, highlighted in shades of blue, was selected for crosses and further characterization. (PDF) [file pgen.1010980.s013.pdf]

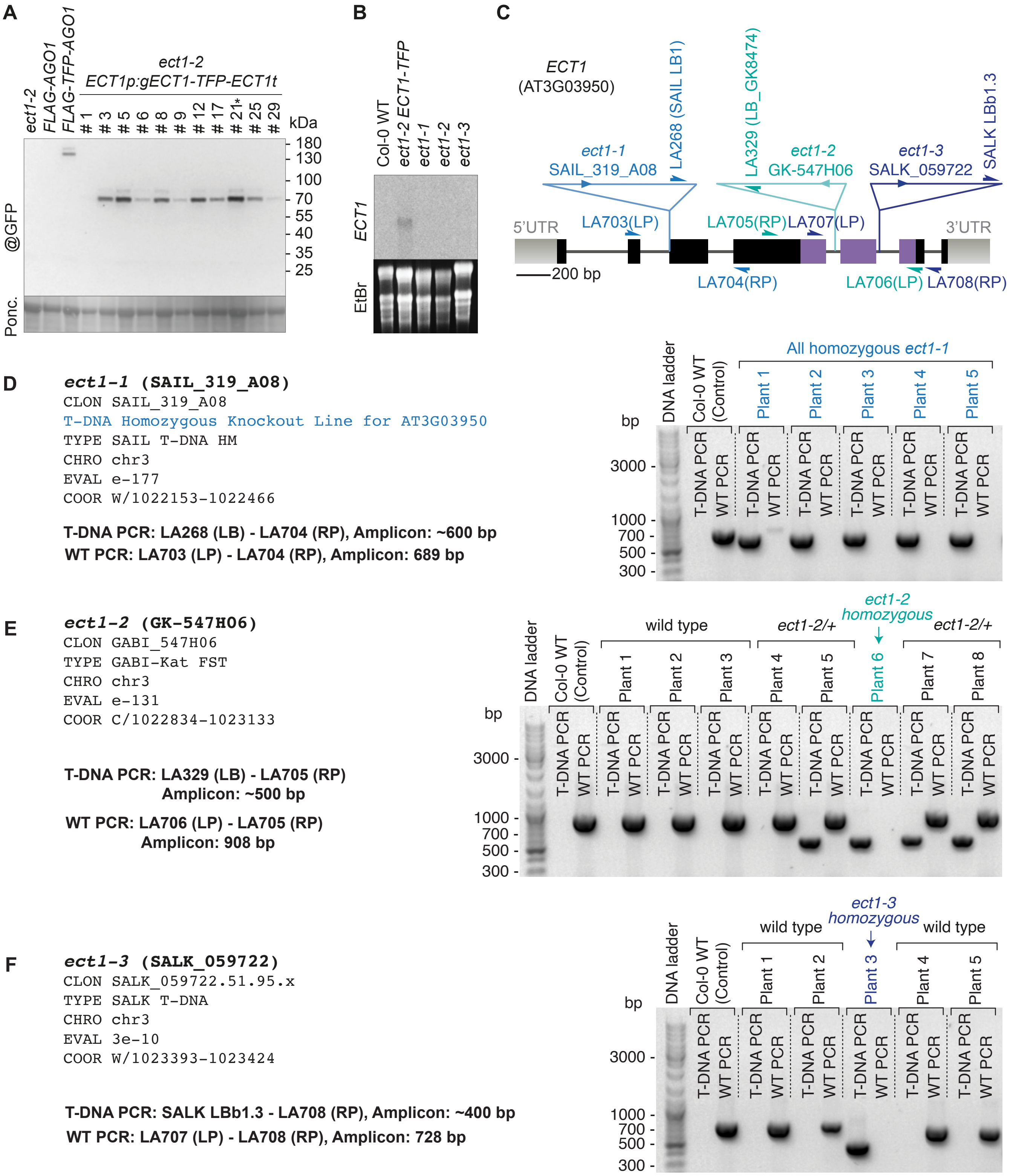

S13 Fig. Isolation of ECT1-TFP transgenic lines and ect1 T-DNA insertion alleles. (A) Western blot using antibodies against GFP (that recognize TFP) in different *ECT1p:gECT1-TFP-ECT1t* independent lines. Ponceau (Ponc.) staining of the membrane is used as loading control. **(B)** Northern blot using the probe (P) specified in Fig 3G to detect *ECT1* mRNA. Although the probe recognizes specifically *ECT1* in the *ECT1p:gECT1-TFP-ECT1t* Line #21 (marked with an asterisk in A), the endogenous expression levels of *ECT1* in Col-0 wild type are below detection limit by northern blot. **(C)** Schematic representation of the *Ath ECT1* locus (At3g03950). Exons are represented as boxes and introns as lines. Untranslated regions (UTRs) are coloured grey, the sequence encoding the YTH domain is purple, and the rest of the *ECT1* coding sequence is black. The IDs and positions of the T-DNA insertions assigned to *ect1-1*, *ect1-2* and *ect1-3* alleles are marked, and so is the location of primers used for their genotyping. **(D-F)** 1% agarose gels showing EtBr-stained PCR fragments corresponding to the genotyping of *ect1-1* (D), *ect1-2* (E) and *ect1-3* (F) in plants germinated from seeds provided by the Nottingham Arabidopsis Stock Center (NASC) as indicated. The primer set for each PCR ('T-DNA' detects the insertion, and 'WT' detects the wild type allele) and the length of the resulting amplicons are indicated to the left of each gel. The sequence of all primers can be found in S2 Table. The progeny of plants homozygous for each T-DNA insertion, highlighted in shades of blue, was selected for crosses and further characterization.
