# Supplementary material for: Insights into the conservation and diversification of the molecular functions of YTHDF proteins
Source: PLoS Genet. 2023 Oct 10;19(10):e1010980. doi: 10.1371/journal.pgen.1010980 (PMC10617740; doi:10.1371/journal.pgen.1010980)
Supplement: S14 Fig — (A) Abbreviations for combinations of double (d), triple (t), or quadruple (q) ect (e) mutants following the nomenclature proposed by Arribas-Hernández et al. [17]. The prefix ‘G’ identifies allele combinations having only T-DNA insertions belonging to the GABI-KAT collection [129]. (B) Morphological appearance of seedlings with or without ECT1 in the different backgrounds indicated, at 9 or 17 days after germination (DAG). (C) Analyses of the percentage of trichomes with 3, 4, 5 or 6 spikes in plants with or without ECT1 in the different backgrounds indicated. n, number of trichomes assessed for each genotype. NS, non-significant differences according to statistical analysis performed as in [16]. (D) Analysis of root growth rate and directionality upon mutation of ECT1 in the ect2-3 and ect2-3/ect3-2 (Gde23) knockout backgrounds. Upper panels represent the overlayed silhouettes of actual roots as they grow on vertically disposed MS-agar plates. n, number of plants assessed for each genotype. VGI, vertical growth index; HGI, horizontal growth index. Root phenotypic analyses were conducted as in [17]. (PDF) [file pgen.1010980.s014.pdf]

A

Short name	Allele combination	First described
Gde12	*ect1-2 ect2-3*	This work
Gde23	*ect2-3 ect3-2*	Arribas-Hernández et al., 2018. The Plant Cell. [16]
Gte123	*ect1-2 ect2-3 ect3-2*	This work
Gte234	*ect2-3 ect3-2 ect4-2*	Arribas-Hernández et al., 2020. Development. [17]
Gqe1234	*ect1-2 ect2-3 ect3-2 ect4-2*	This work
te234	*ect2-1 ect3-1 ect4-2*	Arribas-Hernández et al., 2018. The Plant Cell. [16]
qe1234	*ect1-1 ect2-3 ect3-2 ect4-2*	This work

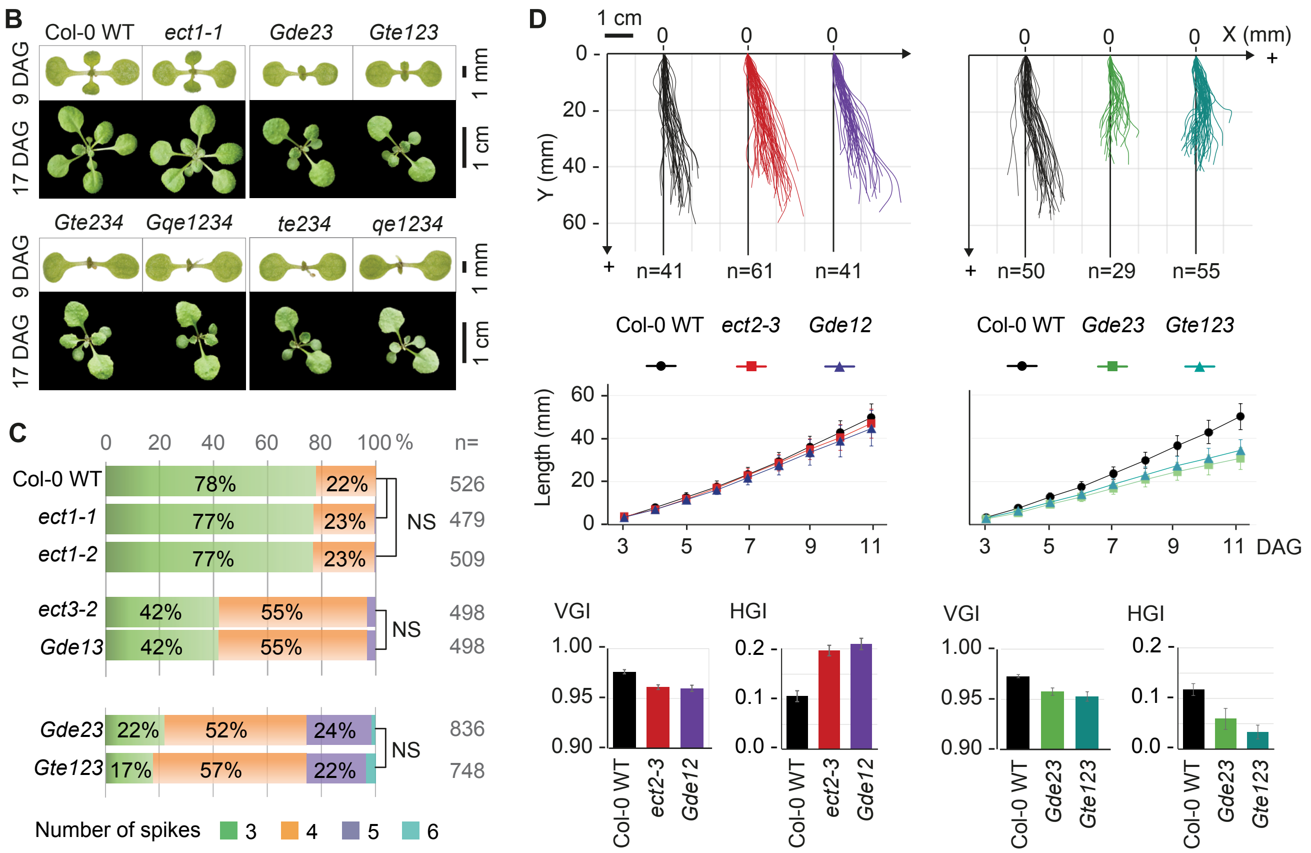

S14 Fig. Knockout of ECT1 does not affect arabidopsis plant development. (A) Abbreviations for combinations of double (d), triple (t), or quadruple (q) ect (e) mutants following the nomenclature proposed by Arribas-Hernández et al. [17]. The prefix ‘G’ identifies allele combinations having only T-DNA insertions belonging to the GABI-KAT collection [127]. **(B)** Morphological appearance of seedlings with or without *ECT1* in the different backgrounds indicated, at 9 or 17 days after germination (DAG). **(C)** Analyses of the percentage of trichomes with 3, 4, 5 or 6 spikes in plants with or without *ECT1* in the different backgrounds indicated. n, number of trichomes assessed for each genotype. NS, non-significant differences according to statistical analysis performed as in [16]. **(D)** Analysis of root growth rate and directionality upon mutation of *ECT1* in the *ect2-3* and *ect2-3/ect3-2* (*Gde23*) knockout backgrounds. Upper panels represent the overlayed silhouettes of actual roots as they grow on vertically disposed MS-agar plates. n, number of plants assessed for each genotype. VGI, vertical growth index; HGI, horizontal growth index. Root phenotypic analyses were conducted as in [17].
