# Supplementary material for: Insights into the conservation and diversification of the molecular functions of YTHDF proteins
Source: PLoS Genet. 2023 Oct 10;19(10):e1010980. doi: 10.1371/journal.pgen.1010980 (PMC10617740; doi:10.1371/journal.pgen.1010980)
Supplement: S15 Fig — Representative 10-day-old te234/US7Yp:cECT(X)IDR-cECT(Y)YTH-mCherry-OCSt T1 seedlings with their fluorescent signal. Bars represent weighed averages of complementation. Scale bars are 1 mm. (PDF) [file pgen.1010980.s015.pdf]

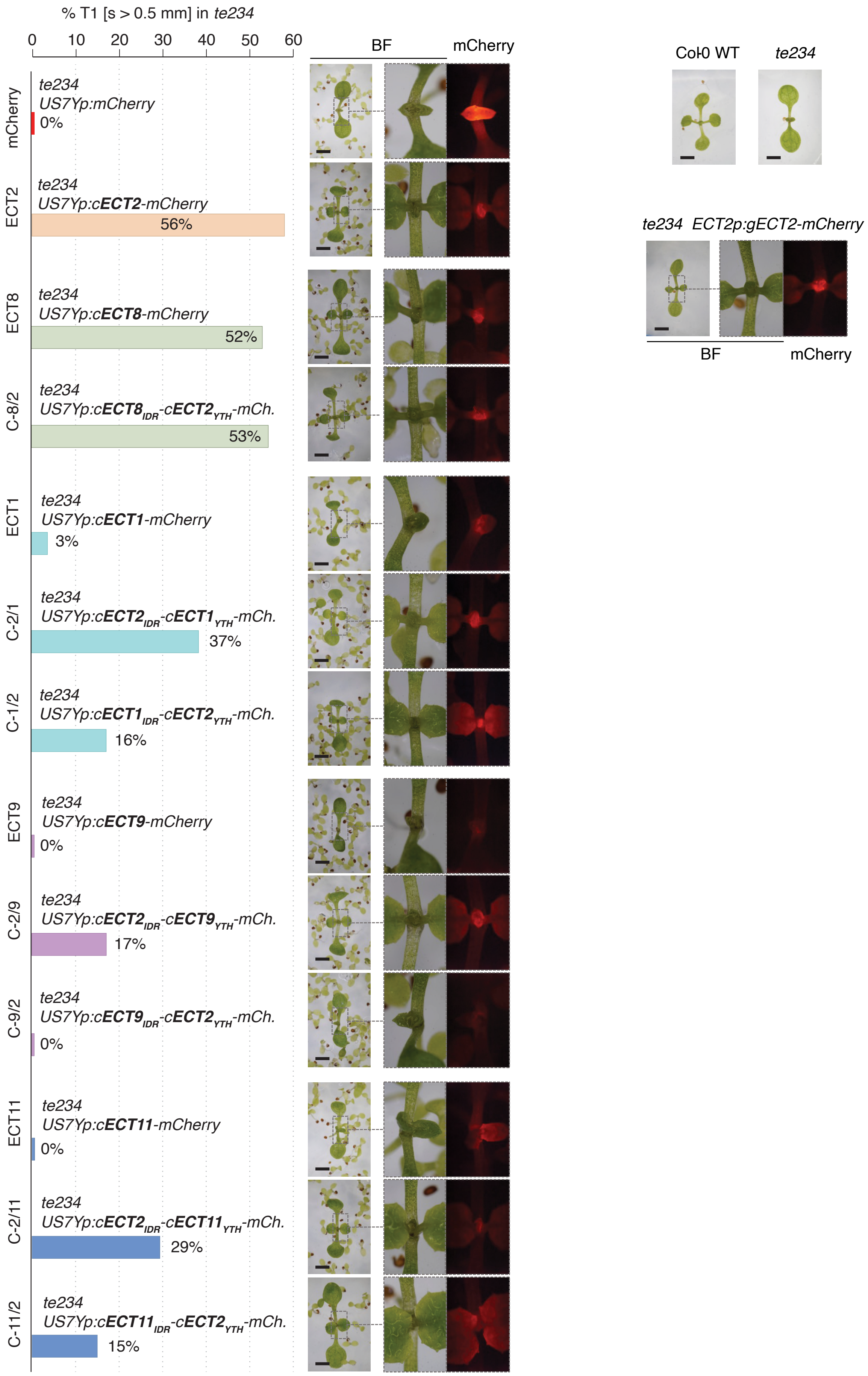

S15 Fig. Expression of chimeric *ECT(X)*$_{IDR}$*-ECT(Y)*$_{YTH}$ constructs in *te234* plants. Representative 10-day-old *te234/uS7Yp:cECT(X)*$_{IDR}$*-cECT(Y)*$_{YTH}$*-mCherry-OCSt* T1 seedlings with their fluorescent signal. Bars represent weighed averages of complementation. Scale bars are 1 mm.
