# Supplementary material for: Insights into the conservation and diversification of the molecular functions of YTHDF proteins
Source: PLoS Genet. 2023 Oct 10;19(10):e1010980. doi: 10.1371/journal.pgen.1010980 (PMC10617740; doi:10.1371/journal.pgen.1010980)
Supplement: S16 Fig — Sequence alignment of the YTH domains of Arabidopsis thaliana (Ath) ECT1-11, Marchantia polymorpha (Mpo) DFE, Saccharomyces cerevisiae (Sc) MRB1/Pho92, Drosophila melanogaster (Dm) YTHDF, and Homo sapiens (Hs) YTHDF2 and YTHDF1. The alignment is colored and annotated using ESPript 3 [130], according to the crystal structure of Hs YTHDF1 in complex with RNA GGm6ACU [61] as template for structural elements. The residues of Hs YTHDF1 that have contacts with RNA are marked above the sequences. Positions in Ath ECT1, ECT9 and ECT11 with non-conservative changes compared to the majority of ECTs are highlighted. Most of these substitutions are located on the protein surface according to 3D homology models of the ECT1/9/11 YTH domains (S17 Fig) except for the following residues: For ECT1, N260 and C261 (α1) are in close proximity to the aromatic cage. For ECT9, N432 (α1) forms hydrogen bonds with m6A. The D to N substitution at that position in all members of the DF-B clade (marked also in ECT5 and ECT10) [26], fern DF-Fs (S4 Fig) and YTHDC proteins [13, 61] increases the affinity for m6A by 15-fold [61] and is likely the product of convergent evolution. The remaining ECT9 substitutions are on the surface of the protein in areas not known to interact with RNA except for S388 (*), also a serine in the DF-B member ECT5 (marked), despite the presence of glycine in this position in most YTHDC and YTHDF proteins of plants, animals and fungi. The backbone NH group of this glycine in Hs YTHDF1 (Gly444) and Zygosaccharomyces rouxii (Zro) MRB1 (Gly233) forms hydrogen bonds, through a water molecule, with the phosphate backbone of the RNA [11,61]. Remarkably, ECT11 contains an arginine (R293) in the same position. Other ECT11 substitutions are on the surface and far from the RNA-binding groove. Of note, plant YTHDF proteins have a small insertion between α2 and β3 that extends α2 further out than in the metazoan orthologs. This insertion is slightly longer in the angiosperm DF-D and [file pgen.1010980.s016.pdf]

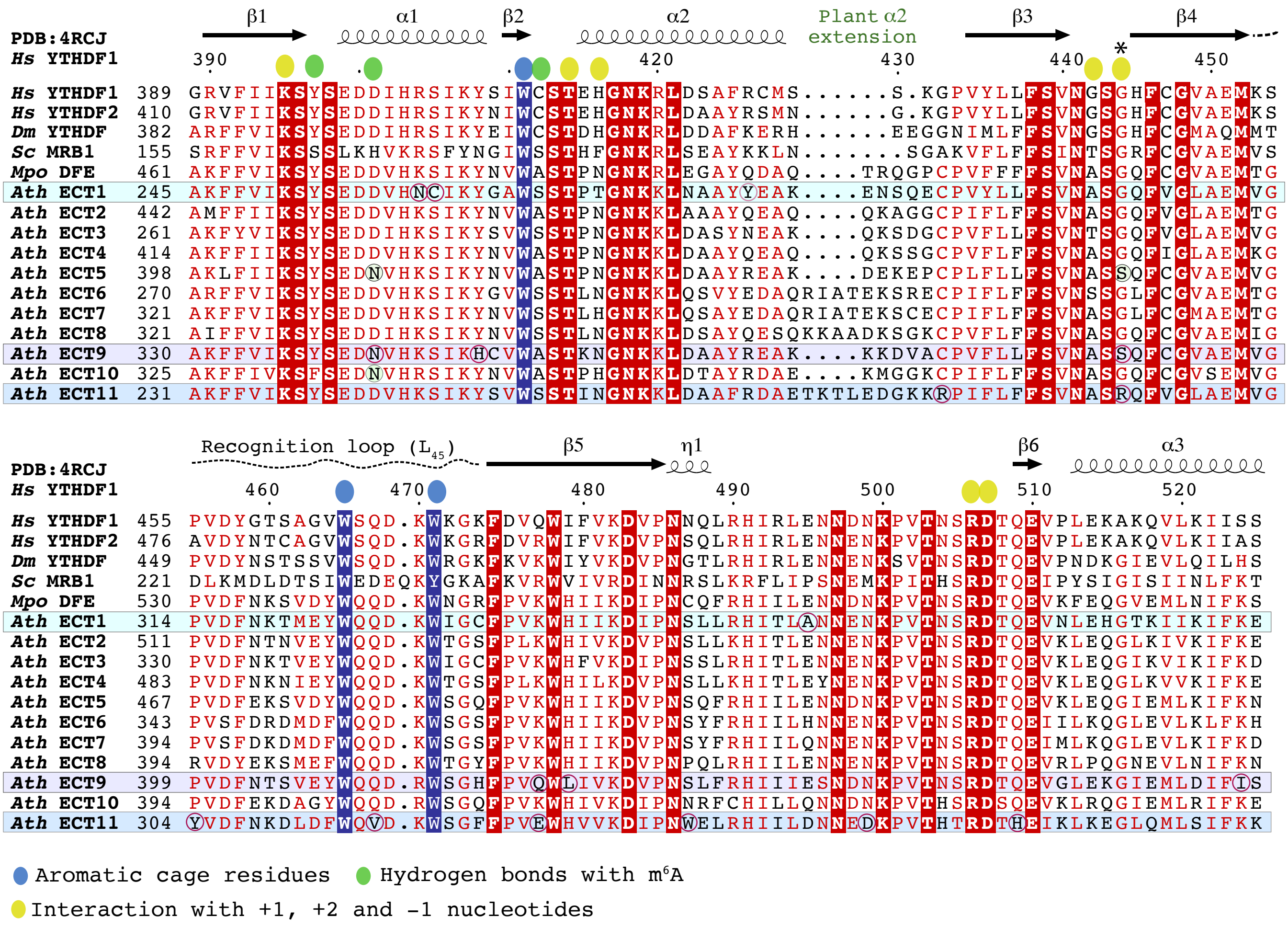


**S16 Fig. Conservation of the YTH domain at the sequence level.** Sequence alignment of the YTH domains of *Arabidopsis thaliana* (*Ath*) ECT1-11, *Marchantia polymorpha* (*Mpo*) DFE, *Saccharomyces cerevisiae* (*Sc*) MRB1/Pho92, *Drosophila melanogaster* (*Dm*) YTHDF, and *Homo sapiens* (*Hs*) YTHDF2 and YTHDF1. The alignment is colored and annotated jusing ESPript 3 [127], according to the crystal structure of *Hs* YTHDF1 in complex with RNA GGm6ACU [61] as template for structural elements. The residues of Hs YTHDF1 that have contacts with RNA are marked above the sequences.
Positions in *Ath* ECT1, ECT9 and ECT11 with non-conservative changes compared to the majority of ECTs are highlighted. Most of these substitutions are located on the protein surface according to 3D homology models of the ECT1/9/11 YTH domains (S17 Fig) except for the following residues: For **ECT1**, N260 and C261 (α1) are in close proximity to the aromatic cage. For **ECT9**, N432 (α1) forms hydrogen bonds with $m^6A$. The D to N substitution at that position in all members of the DF-B clade (marked also in ECT5 and ECT10) [26], fern DF-Fs (S4 Fig) and YTHDC proteins [13, 61] increases the affinity for $m^6A$ by 15-fold [61] and is likely the product of convergent evolution. The remaining ECT9 substitutions are on the surface of the protein in areas not known to interact with RNA except for S388 (*), also a serine in the DF-B member ECT5 (marked) despite the presence of glycine in this position in most YTHDC and YTHDF proteins of plants, animals and fungi. The backbone NH group of this glycine in *Hs* YTHDF1 (Gly444) and *Zygosaccharomyces rouxii* (*Zro*) MRB1 (Gly233) forms hydrogen bonds, through a water molecule, with the phosphate backbone of the RNA [11, 61]. Remarkably, **ECT11** contains an arginine (R293) in the same position. Other ECT11 substitutions are on the surface and far from the RNA-binding groove.
Of note, plant YTHDF proteins have a small insertion between α2 and β3 that extends α2 further out than in the metazoan orthologs. This insertion is slightly longer in the angiosperm DF-D and -C clades, which include *Ath* ECT11 (S17 Fig), as well as in all members of the fern/gymnosperm-exclusive DF-F clade (S4 Fig), and in many bryophyte and lycophyte DF-Es (S4 Fig).
