# Supplementary material for: Insights into the conservation and diversification of the molecular functions of YTHDF proteins
Source: PLoS Genet. 2023 Oct 10;19(10):e1010980. doi: 10.1371/journal.pgen.1010980 (PMC10617740; doi:10.1371/journal.pgen.1010980)
Supplement: S17 Fig — Three left panels, AlphaFold-predicted [74,75] apo structures of YTH domains of Arabidopsis thaliana (Ath) ECT1/9/11 in comparison to that of Ath ECT2, and of Homo sapiens (Hs) YTHDF1. Red/blue coloring in the left-most panel indicates the electrostatic potential of the surface calculated using the APBS PyMOL plugin [131] with the parameters described in Methods. The two middle panels show the similarity of the secondary structure of all proteins and highlight the few non-conservative amino acid substitutions identified in Ath ECT1/9/11 (S16 Fig). Right panel, hydrophobic cage of the same proteins modelled on the crystal structure of the YTH domain of Hs YTHDF1 in complex with RNA GGm6ACU [61] (Protein Data Bank entry 4RCJ). The key residues for m6A binding are marked, including the aspartic to asparagine substitution in the DF-B clade member Ath ECT9 (N342). (PDF) [file pgen.1010980.s017.pdf]

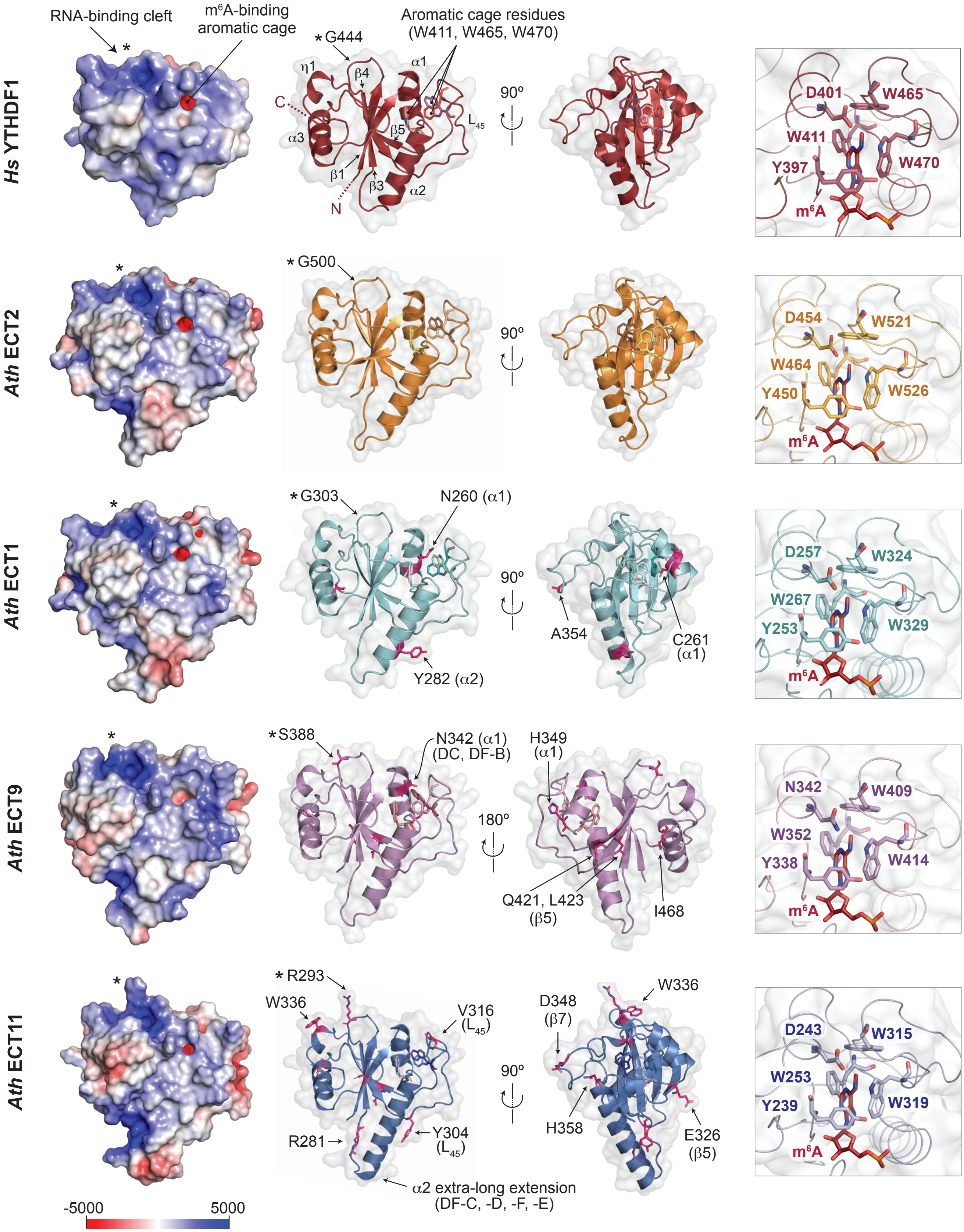

S17 Fig. Conservation of the YTH domain at the structural level. Three left panels, AlphaFold-predicted [73, 74] apo structures of YTH domains of *Arabidopsis thaliana* (*Ath*) ECT1/9/11 in comparison to that of *Ath* ECT2, and of *Homo sapiens* (*Hs*) YTHDF1. Red/blue coloring in the left-most panel indicates the electrostatic potential of the surface calculated using the APBS PyMOL plugin [128] with the parameters described in Methods. The two middle panels show the similarity of the secondary structure of all proteins and highlight the few non-conservative amino acid substitutions identified in *Ath* ECT1/9/11 (S16 Fig). Right panel, hydrophobic cage of the same proteins modelled on the crystal structure of the YTH domain of *Hs* YTHDF1 in complex with RNA GGm^{6}ACU [61] (Protein Data Bank entry 4RCJ). The key residues for m^{6}A binding are marked, including the aspartic to asparagine substitution in the DF-B clade member *Ath* ECT9 (N342).
