# Supplementary material for: Insights into the conservation and diversification of the molecular functions of YTHDF proteins
Source: PLoS Genet. 2023 Oct 10;19(10):e1010980. doi: 10.1371/journal.pgen.1010980 (PMC10617740; doi:10.1371/journal.pgen.1010980)
Supplement: S18 Fig — Left columns: Structure of Arabidopsis thaliana (Ath) ECT proteins predicted by AlphaFold [74,75] colored according to a confidence score (pLDDT) given per-residue between 0 and 100. Regions below 50 pLDDT may be unstructured in isolation [75, 132]. The vast majority of N-terminal regions in ECT proteins score below 50 pLDDT (dark orange). A few short stretches with low-confidence prediction of secondary structure are marked with black arrows for ECT1/9/11. Middle column: Disorder prediction by MobiDB [77] depicted by red (disorder) or light blue (structure) coloring of the AlphaFold-predicted structure. The N-terminal regions of all ECT proteins are predicted to be largely disordered (red). Right columns, sequences and linear representation of the proteins indicating MobiDB [77] disorder predictions (‘Disorder’ track) with the same colors as in the middle column. The known structure of the YTH domain of Homo sapiens (Hs) YTHDF2 is marked in dark blue, and the YTH domains of Ath ECTs, with no experimentally resolved structures to date, have been manually added also in dark blue. Purple bars superimposed on the amino acid sequences of the proteins and purple boxes positioned along the linear representations on the ‘Linear interacting peptide’ (LIP) tracks highlight residues predicted to interact with another molecule that preserve structural linearity in the bound state. In addition to LIPs, they are generally called short linear motifs (SLiMs), molecular recognition features (MoRFs) or protean segments (ProS). The UniProt ID of all proteins is given below their names. (PDF) [file pgen.1010980.s018.pdf]

Alphafold pLDDT score
(Model confidence)

- Very high (pLDDT > 90)
- Confident (90 > pLDDT > 70)
- Low (70 > pLDDT > 50)
- Very low (pLDDT < 50)

MobiDB prediction

- Observed
- Structure
- Disorder

MobiDB prediction of:
- Structure: red (disorder) or blue (structure).
- Linear interacting peptides (LIPs): purple bars along the linear representation and superimposed on the sequence.

Hs YTHDF2
(Q9Y5A9)

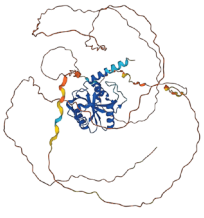

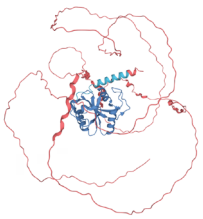

Hs YTHDF2

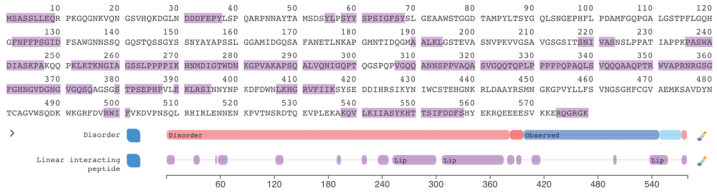

ECT1
(Q3MK94)

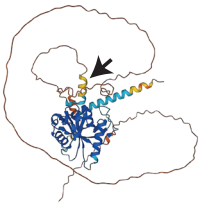

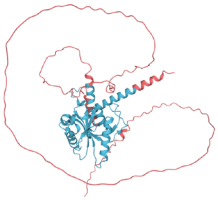

ECT1

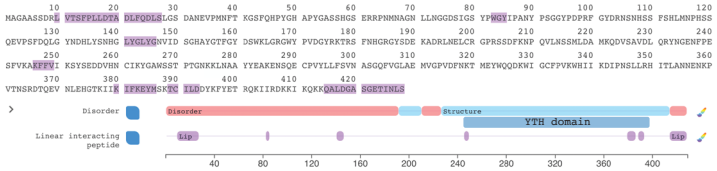

ECT2
(Q9LJE5)

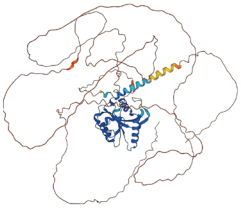

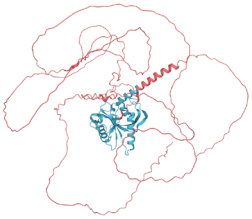

ECT2

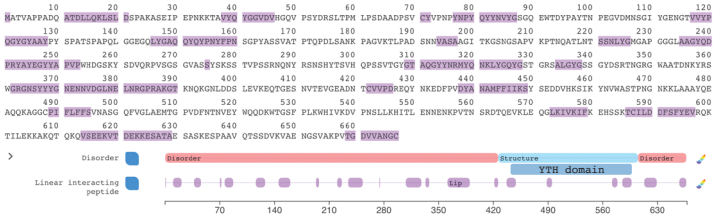

ECT3
(F4K1Z0)

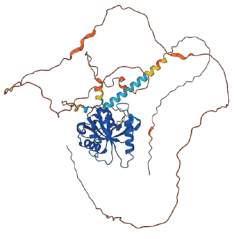

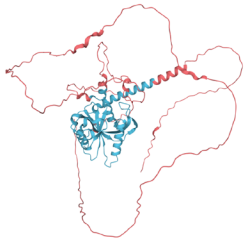

ECT3

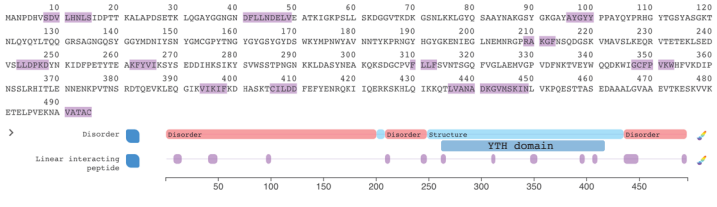

ECT4
(A0A1P8AS03)

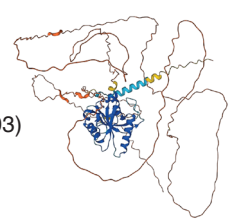

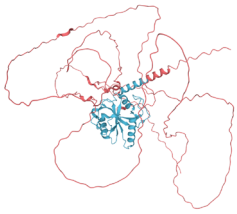

ECT4

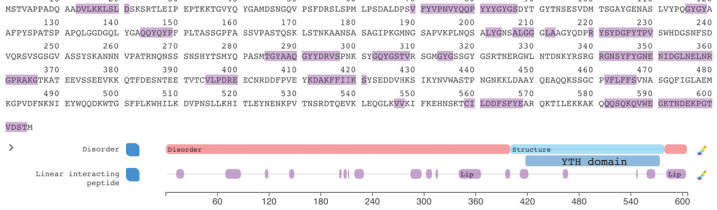

ECT5
(Q0WR25)

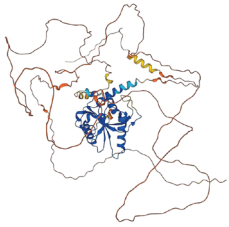

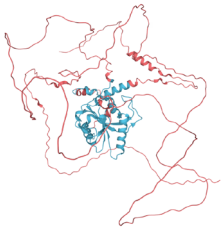

ECT5

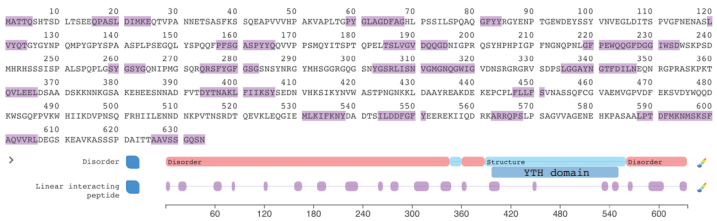

ECT6
(Q1JPL5)

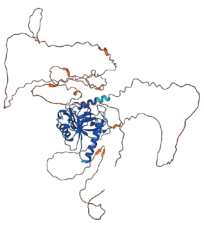

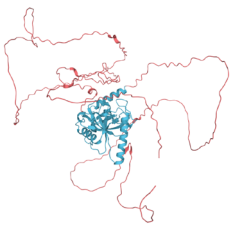

ECT6

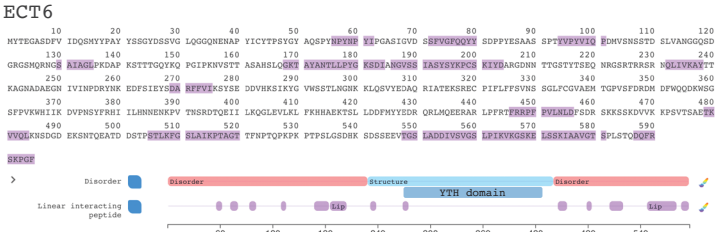

S18 Fig, continues on the next page

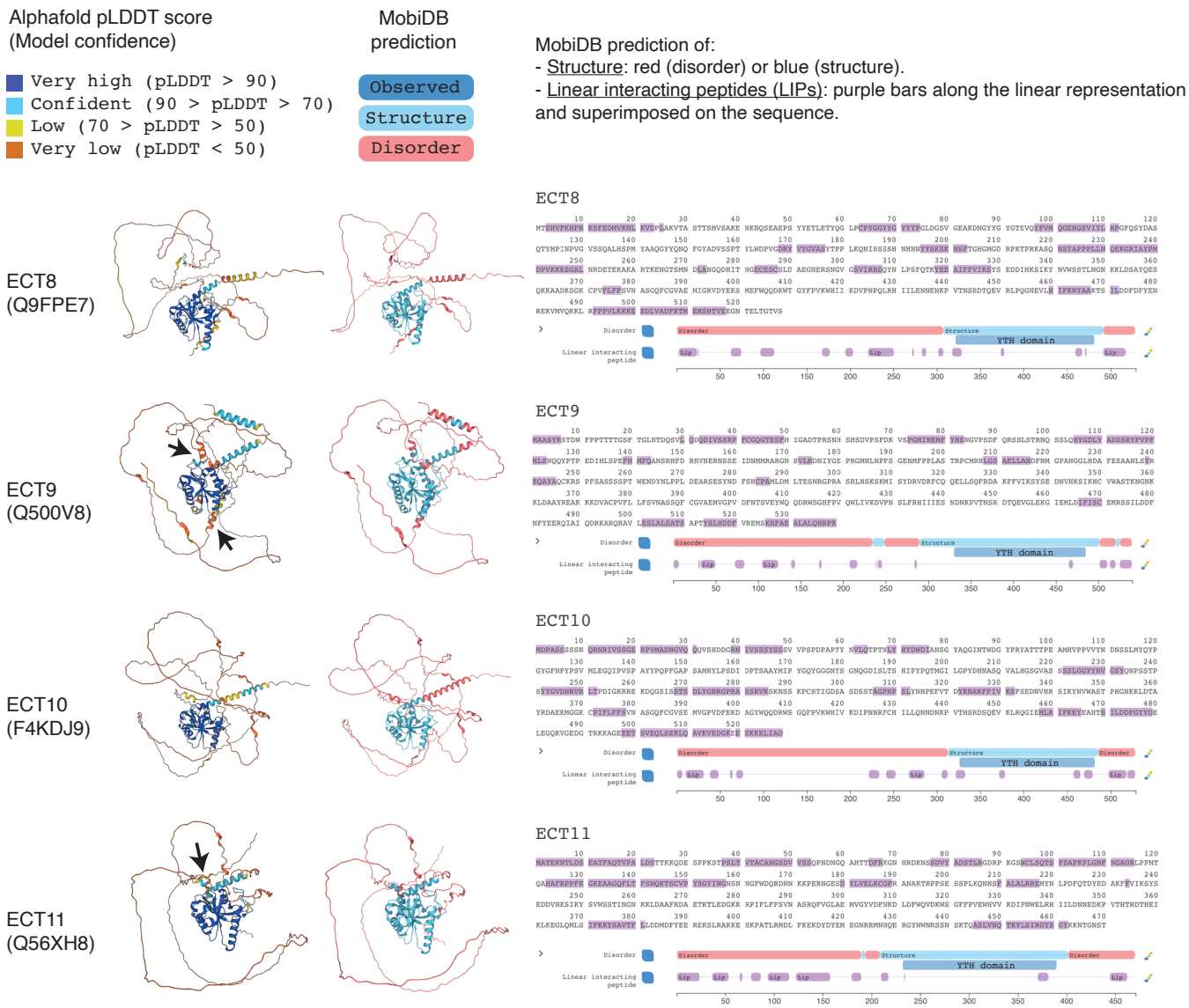

S18 Fig. Predicted structure and short linear motifs (SLiMs) of arabidopsis ECTs compared to human YTHDF2. Left columns: Structure of *Arabidopsis thaliana* (*Ath*) ECT proteins predicted by AlphaFold [73, 74] colored according to a confidence score (pLDDT) given per-residue between 0 and 100. Regions below 50 pLDDT may be unstructured in isolation [74, 129]. The vast majority of N-terminal regions in ECT proteins score below 50 pLDDT (dark orange). A few short stretches with low-confidence prediction of secondary structure are marked with black arrows for ECT1/9/11. Middle column: Disorder prediction by MobiDB [76] depicted by red (disorder) or light blue (structure) coloring of the AlphaFold-predicted structure. The N-terminal regions of all ECT proteins are predicted to be largely disordered (red). **Right columns**, sequences and linear representation of the proteins indicating MobiDB [76] disorder predictions ('Disorder' track) with the same colors as in the middle column. The known structure of the YTH domain of *Homo sapiens* (*Hs*) YTHDF2 is marked in dark blue, and the YTH domains of *Ath* ECTs, with no experimentally resolved structures to date, have been manually added also in dark blue. Purple bars superimposed on the amino acid sequences of the proteins and purple boxes positioned along the linear representations on the 'Linear interacting peptide' (LIP) tracks highlight residues predicted to interact with another molecule that preserve structural linearity in the bound state. In addition to LIPs, they are generally called short linear motifs (SLiMs), molecular recognition features (MoRFs) or protean segments (ProS). The UniProt ID of all proteins is given below their names.
