# Supplementary material for: Insights into the conservation and diversification of the molecular functions of YTHDF proteins
Source: PLoS Genet. 2023 Oct 10;19(10):e1010980. doi: 10.1371/journal.pgen.1010980 (PMC10617740; doi:10.1371/journal.pgen.1010980)
Supplement: S19 Fig — (A) Time-averaged density profiles of the slab simulations of the ECT proteins shown in Fig 5B. (B) Relationship between excess transfer free energy from dilute to dense phase (ΔGtrans = RT ln [cdilute / cdense]) and average stickiness (λ) of the IDR residues. (C-D) Comparative amino acid composition (C) and representation of charges (D) along the IDR sequences of the different YTHDF proteins assayed in this study. In D, arrows highlight the overall trends in charge change along the IDRs. Notice that the length in amino acids (aa) is not at scale to simplify the representation. (PDF) [file pgen.1010980.s019.pdf]

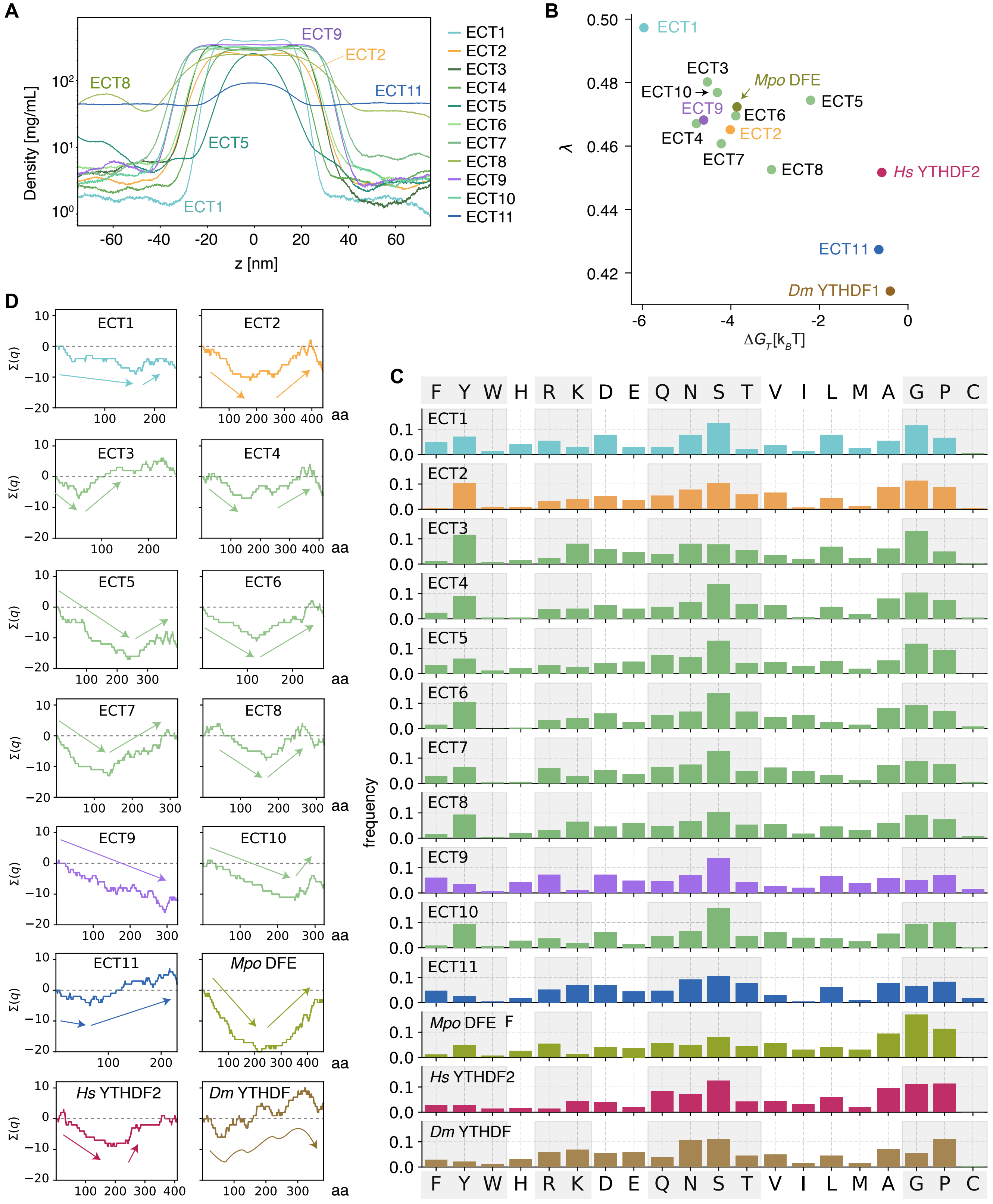

S19 Fig. Analysis of the IDRs of the YTHDF proteins assayed in this study (extended data). (A) Time-averaged density profiles of the slab simulations of the ECT proteins shown in Fig 5B. **(B)** Relationship between excess transfer free energy from dilute to dense phase (ΔG_{trans} = RT ln (c_{dilute} / c_{dense})) and average stickiness (λ) of the IDR residues. **(C-D)** Comparative amino acid composition (C) and representation of charges (D) along the IDR sequences of the different YTHDF proteins assayed in this study. In D, arrows highlight the overall trends in charge change along the IDRs. Notice that the length in amino acids (aa) is not at scale to simplify the representation.
