# Supplementary material for: Insights into the conservation and diversification of the molecular functions of YTHDF proteins
Source: PLoS Genet. 2023 Oct 10;19(10):e1010980. doi: 10.1371/journal.pgen.1010980 (PMC10617740; doi:10.1371/journal.pgen.1010980)

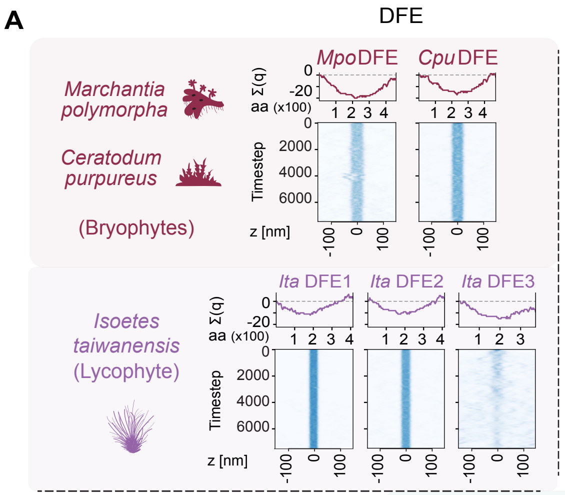

S21 Fig. Analysis of the IDRs of YTHDF proteins from representative species of the main taxa of land plants. (A) Representation of charges (top panels) and slab simulations (bottom panels) of the IDRs of YTHDF proteins from the main taxa of land plants. The proteins are sorted and the charge distributions coloured according to species and land plant evolution from top (basal group) to bottom, and the different plant DF clades are separated along the horizontal axis. *Isoetes taiwanensis* (*Ita*) DF-CD (Fig 1B) is excluded from the analysis because its N-terminus is very short (S1 Dataset) and does not behave like an IDR. **(B)** Relationship between excess transfer free energy from dilute to dense phase ($\Delta G_{trans} = RT \ln [c_{dilute} / c_{dense}]$) and average stickiness (λ) of the IDR residues of the proteins in A. The proteins are color-coded according to plant DF clades as indicated. *Homo sapiens* (*Hs*) YTHDF2 is included as a reference. Same as for the *Arabidopsis thaliana* (*Ath*) ECT set in S19B Fig, the content of sticky residues defined by the CALVADOS model [78] mainly governs the different phase separation propensity among the IDRs. A clade-dependent separation of the proteins according to these properties is apparent.

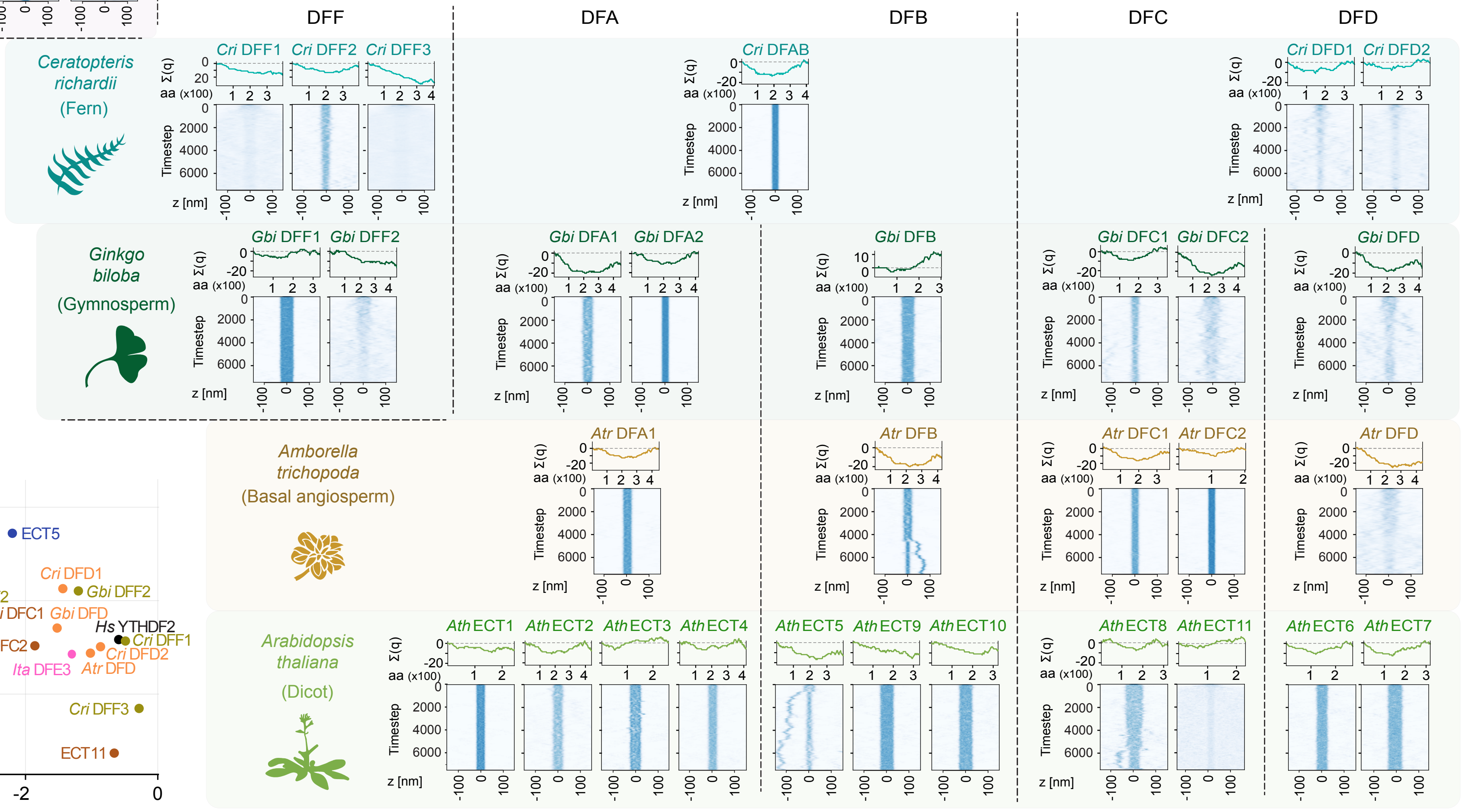

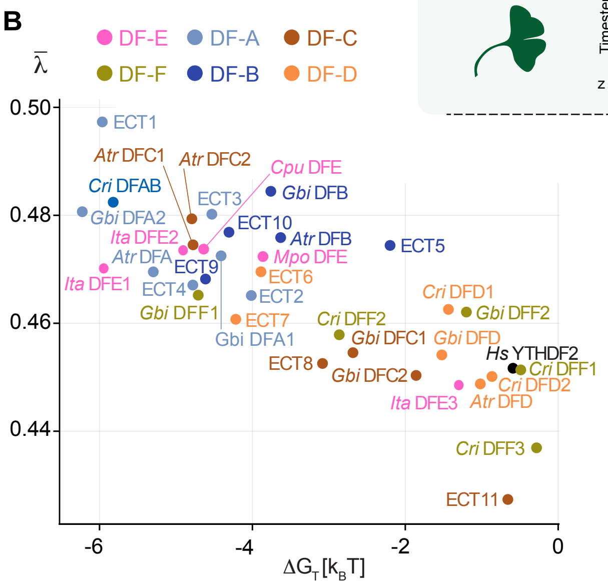

Supplement: S21 Fig — (A) Representation of charges (top panels) and slab simulations (bottom panels) of the IDR sequences of YTHDF proteins from the main taxa of land plants. The proteins are sorted according to land plant evolution (vertical axis) and plant DF clades (horizontal axis). Isoetes taiwanensis (Ita) DF-CD (Fig 1B) is excluded from the analysis because its N-terminus is very short (S1 Dataset) and does not behave like an IDR. (B) Relationship between excess transfer free energy from dilute to dense phase (ΔGtrans = RT ln [cdilute / cdense]) and average stickiness (λ) of the IDR residues of the proteins in A. The proteins are color-coded according to plant DF clades. Homo sapiens (Hs) YTHDF2 is included as a reference. Same as for the Arabidopsis thaliana (Ath) ECT set in S19B Fig, the content of sticky residues defined by the CALVADOS model [78] mainly governs the different phase separation propensity among the IDRs. A clade-dependent separation of the proteins according to these properties is apparent. (PDF) [file pgen.1010980.s021.pdf]
