# Supplementary material for: Insights into the conservation and diversification of the molecular functions of YTHDF proteins
Source: PLoS Genet. 2023 Oct 10;19(10):e1010980. doi: 10.1371/journal.pgen.1010980 (PMC10617740; doi:10.1371/journal.pgen.1010980)
Supplement: S22 Fig — (A-C) Prediction of domains and other features in the protein sequences of Kni DF1-3 according to InterPro (former Pfam) (https://www.ebi.ac.uk/interpro/) [133]. Horizontal axes indicate protein length in amino acids (aa). InterPro entries (unique protein homologous superfamilies, families, domains, repeats or important sites based on one or more signatures) are indicated on the right side. Disorder prediction is engineered by MobiDB (https://mobidb.bio.unipd.it/) [77], and transmembrane domains are predicted by Phobius (https://phobius.sbc.su.se/) [134]. For Kni DF2 in B, the graphical output of Phobius transmembrane topology predictor is shown. (PDF) [file pgen.1010980.s022.pdf]

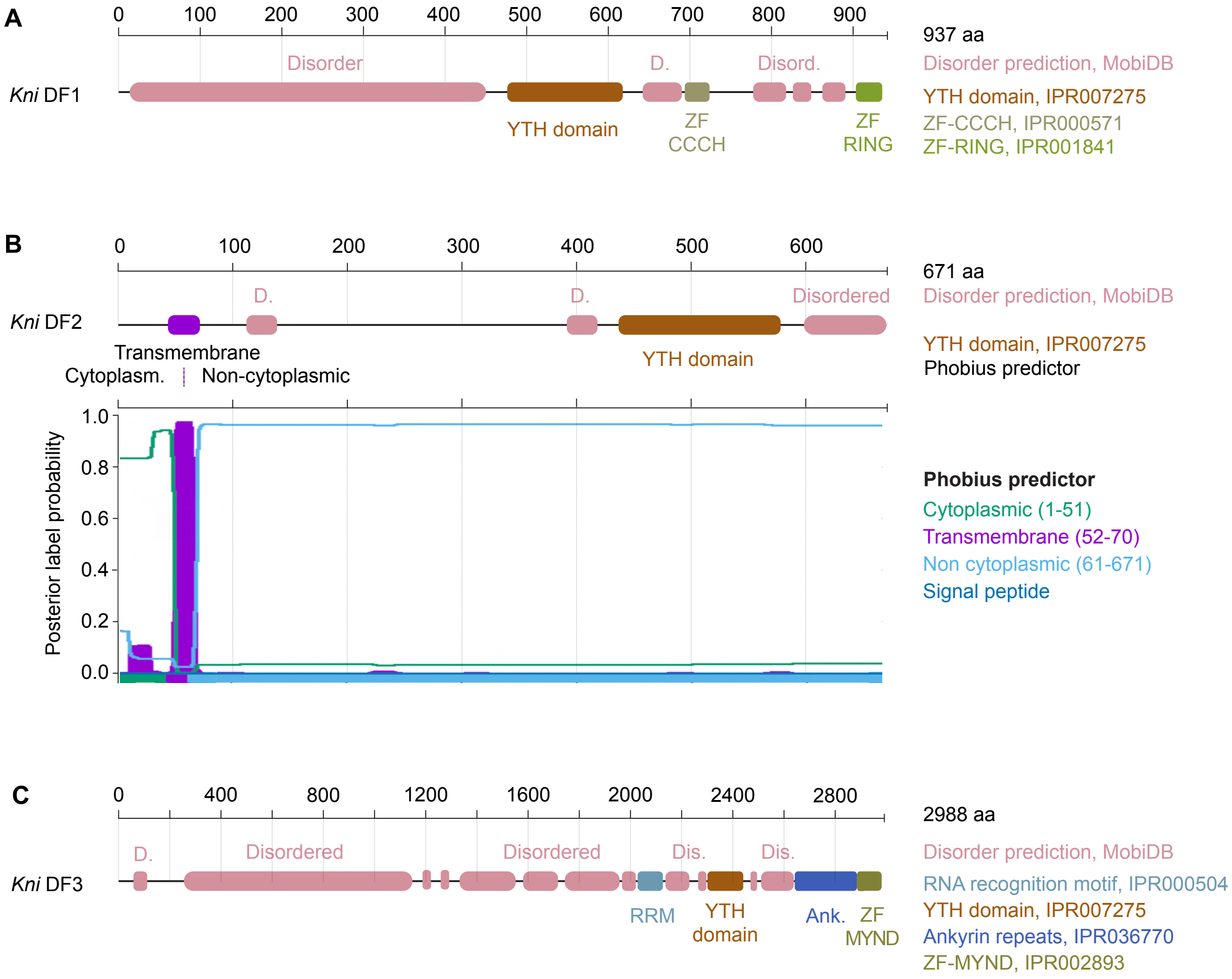

S22 Fig. Analysis of domain composition of YTHDF proteins from the charophyte *Klebsormidium nitens* (*Kni*). (A-C) Prediction of domains and other features in the protein sequences of *Kni* DF1-3 according to InterPro (former Pfam) (https://www.ebi.ac.uk/interpro/) [130]. Horizontal axes indicate protein length in amino acids (aa). InterPro entries (unique protein homologous superfamilies, families, domains, repeats or important sites based on one or more signatures) are indicated on the right side. Disorder prediction is engineered by MobiDB (https://mobidb.bio.unipd.it/) [76], and transmembrane domains are predicted by Phobius (https://phobius.sbc.su.se/) [131]. For *Kni* DF2 in B, the direct graphical output of Phobius Transmembrane Topology Predictor is shown.
