# Supplementary material for: Insights into the conservation and diversification of the molecular functions of YTHDF proteins
Source: PLoS Genet. 2023 Oct 10;19(10):e1010980. doi: 10.1371/journal.pgen.1010980 (PMC10617740; doi:10.1371/journal.pgen.1010980)
Supplement: S23 Fig — (A-B) Overview of the full-length amino acid sequence alignment of Viridiplantae YTHDF proteins (S2 Dataset) (A), and magnification of the trimmed region used for phylogenetic analyses (S3 Dataset) (B). The conservation score provided by Jalview [114] is shown below both panels, and the threshold used for trimming is marked with a horizontal dotted line in A. Blue-white colouring reflects percentage identity according to Jalview [114], with more intense shades of blue for the most highly conserved residues. Grey indicates gaps. In A, the trimmed region is coloured green, with a darker shade highlighting the position of the canonical YTH domain [8]. In B, the YTH domain is framed with a dashed dark green outline, and the aromatic residues that conform the m6A-recognition cage are coloured red and marked with red arrows. (PDF) [file pgen.1010980.s023.pdf]

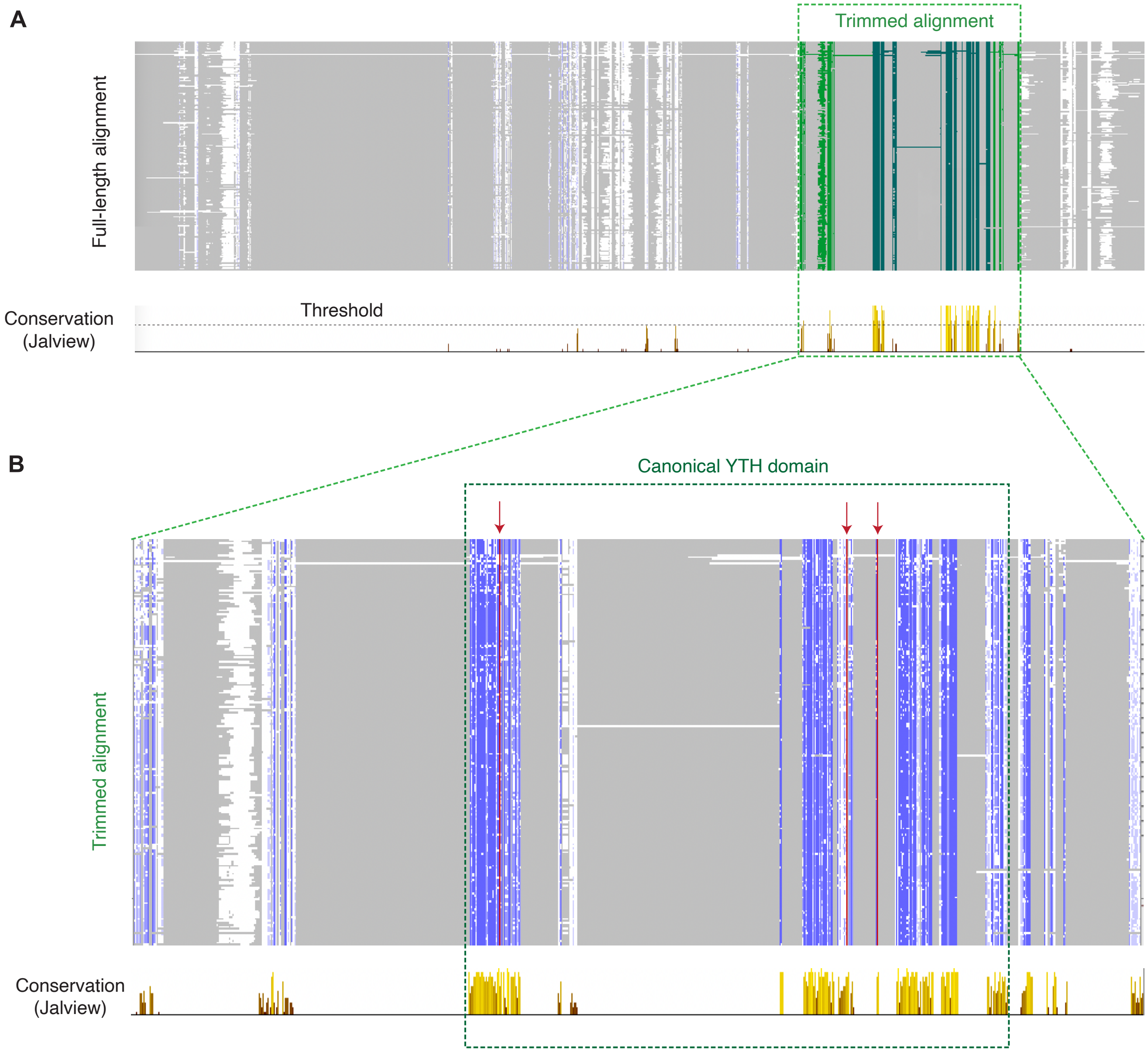

S23 Fig. Trimming of the alignment of Viridiplantae YTHDF proteins according to conservation for phylogenetic analyses. (A-B) Overview of the full-length amino acid sequence alignment of Viridiplantae YTHDF proteins (S2 Dataset) (A), and magnification of the trimmed region used for phylogenetic analyses (S3 Dataset) (B). The conservation score provided by Jalview [112] is shown below both panels, and the threshold used for trimming is marked with a horizontal dotted line in A. Blue-white colouring reflects percentage identity according to Jalview [112], with more intense shades of blue for the most highly conserved residues. Grey indicates gaps. In A, the trimmed region is coloured green, with a darker shade highlighting the position of the canonical YTH domain [8]. In B, the YTH domain is framed with a dashed dark green outline, and the aromatic residues that conform the m^6A-recognition cage are coloured red and marked with red arrows.
