# Supplementary material for: Insights into the conservation and diversification of the molecular functions of YTHDF proteins
Source: PLoS Genet. 2023 Oct 10;19(10):e1010980. doi: 10.1371/journal.pgen.1010980 (PMC10617740; doi:10.1371/journal.pgen.1010980)
Supplement: S20 Fig — (A) T1 plants expressing the long or short splice forms of Marchantia polymorpha (Mpo) DFE 19 days after germination (DAG). Additional genotypes are included as a reference. (B) Morphological appearance and fluorescence pattern of T2 plants expressing Mpo DFEL (L, long isoform) or Homo sapiens (Hs) YTHDF2 in the te234 background 9 or 17 DAG. Additional genotypes are included as a reference. For Hs YTHDF2, several plants are shown to reflect the variability in phenotypic defects, which include enhanced aberrance in the shape or number of cotyledons and first true leaves, and slower overall growth. C, cotyledons; L, true leaves. White scale bars are 1 cm, and black are 1 mm. (PDF) [file pgen.1010980.s020.pdf]

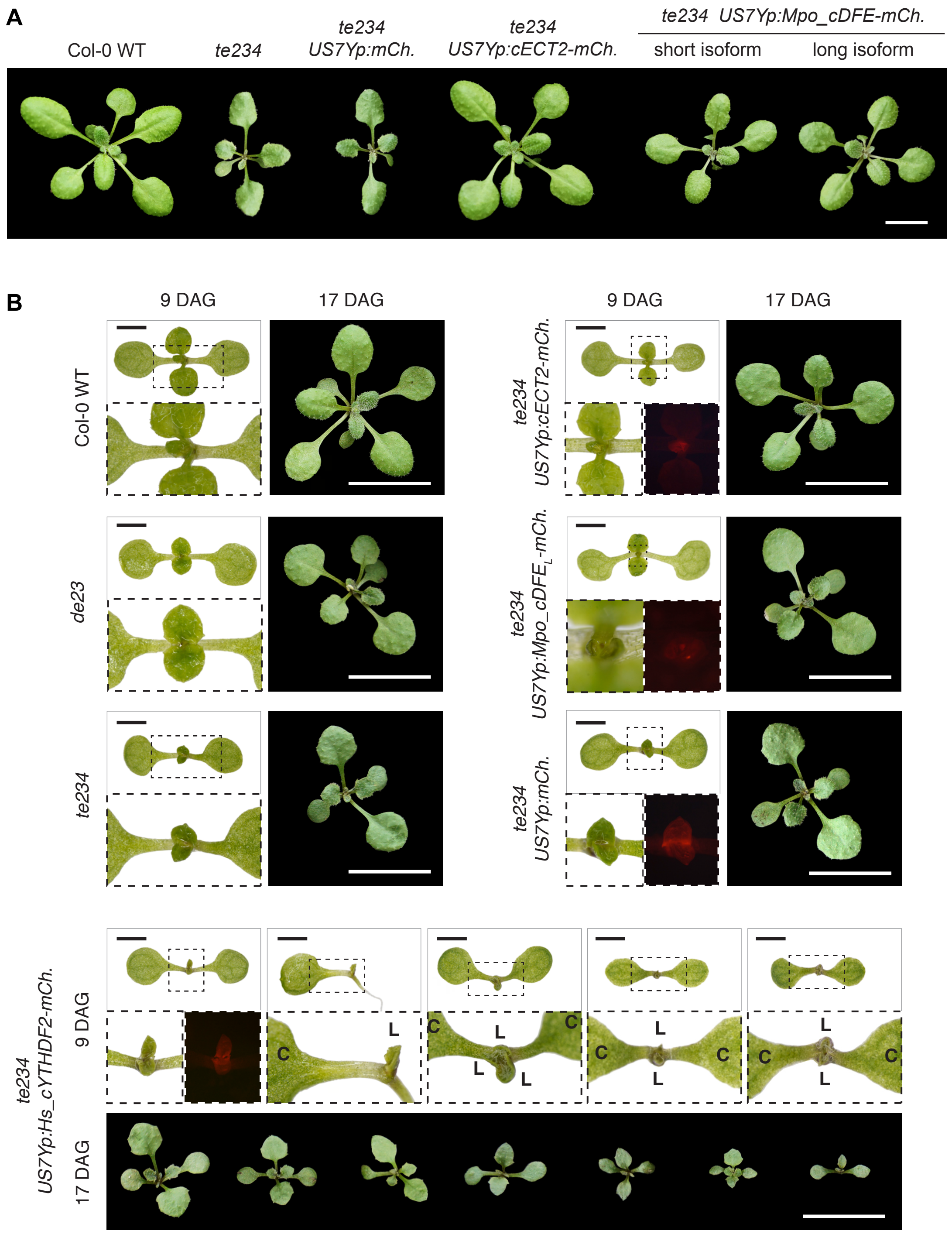

S20 Fig. Heterologous expression of bryophyte and metazoan YTHDF proteins in arabidopsis (extended data). (A) T1 plants expressing the long or short splice forms of *Marchantia polymorpha* (*Mpo*) DFE 19 days after germination (DAG). Additional genotypes are included as a reference. **(B)** Morphological appearance and fluorescence pattern of T2 plants expressing *Mpo* DFE$_L$ (L, long isoform) or *Homo sapiens* (*Hs*) YTHDF2 in the *te234* background 9 or 17 DAG. Additional genotypes are included as a reference. For *Hs* YTHDF2, several plants are shown to reflect the variability in phenotypic defects, which include enhanced aberrance in the shape or number of cotyledons and first true leaves, and slower overall growth. C, cotyledons; L, true leaves. White scale bars are 1 cm, and black are 1 mm.
