# Supplementary material for: Insights into the conservation and diversification of the molecular functions of YTHDF proteins
Source: PLoS Genet. 2023 Oct 10;19(10):e1010980. doi: 10.1371/journal.pgen.1010980 (PMC10617740; doi:10.1371/journal.pgen.1010980)
Supplement: Attachment — Submitted filename: Response to reviewers.pdf [file pgen.1010980.s040.pdf]

RESPONSE TO REVIEWERS

We thank all three reviewers for evaluating our work and explaining to us what they see as strengths and weaknesses. We have seriously considered all points raised and are now submitting a substantially improved version. Major changes and additions focus on:

(i) the phylogenetic analyses that are now much more comprehensive compared to the originally submitted version (please see Response to Reviewer 3),

(ii) analysis of IDRs from additional species representing bryophytes, lycophytes, ferns and gymnosperms,

(iii) rewriting the Discussion so that it now achieves two important points compared to the original version. It now clarifies the areas where the study actually does provide new mechanistic insight, and, in particular, it has a much more comprehensive and interesting treatment of when the ancestral plant YTHDF function in growth stimulation arose, incorporating sequence data and analyses on green algal YTHDFs in the discussion.

We note that for a few points, we are unclear on what exactly the reviewers are criticizing, and on a few others, we disagree with the points raised. All of these cases concern points raised only by a single reviewer, and where at least one other reviewer expresses a sentiment opposite to the criticism raised. We have taken no action to change the manuscript on such points, but benefit from this letter to thoroughly and respectfully explain why this has been our choice in those few cases.

We hope that the reviewers appreciate the improvements and the effort that went into bringing them about, and that they now find our study to be of a quality appropriate for publication.

Reviewer #1: Adenosine methylation in mRNA is known to play important roles in plant development, and the YTH (plant ECT) family of proteins are known to be the main “readers” that bind to RNAs containing m6A. However, much confusion and lack of information exists around how the binding of such proteins brings about normal growth. The eukaryote YTH family can be further divided into nuclear YTHDC-type and cytoplasmic YTHDF-type readers. In both plants and animals, it is the YTHDF-type that have been evidenced as being most critical for bringing about the majority of the m6A-dependent post transcriptional outcomes. In humans there are three YTHDFs, and roles in promoting mRNA translation, degradation or stabilization have variously been claimed, though there is a growing consensus that all promote destabilisation. Higher land plants have an expanded family of YTHDFs, and determining whether these all carry out the same function or have different (perhaps even opposing) functions is a pressing question in the field, which this paper seeks to address.

Overall, the manuscript is very well written, the experiments are clear and easy to follow and there are several important findings that will be of considerable interest to the field. Most notably;

1) The fact that the single YTHDF found in liverworts can rescue the ECT2/3/4 phenotype is a critical finding as it indicates a conserved and ancient function for basal plant YTHDF function.

2) The finding that ECT1 does not rescue ECT2/3/4 is unexpected and appears to be primarily due to altered functionality of the disordered N terminus. This might have been due to more complex regulation of ECT1, such as requirement for post translational modification. However, this is argued against by the lack of additional growth defect in the quadruple mutant.

3) The complementation by at least some members of each of the 4 ECT sub groups suggests that the primordial function has been retained. This suggests that the reason for expansion of this family in plants may reflect differences in expression/regulation of the different ECTs rather than evolution of different functions upon mRNA binding. However, the lack of complementation by ECT9, ECT1 and ECT11 is curious. The elegant domain swap experiments indicate that this is likely due to neofunctionalisation of the disordered domains.

4) The enhancement of the triple mutant phenotype following expression of ECT9 is particularly striking, especially as this seems to be dependent upon the presence of the existing te234 mutations. This may suggest an antagonistic function in this case and this is likely to become an active area of research.

The human YTHDF2 also enhanced the te234 phenotype - likely due to competition for m6A binding, but remains an interesting and potentially useful observation.

5) The combination of complementation experiments, domain swap-complementation and (in the case of ECT9) inhibition, taken together with the known requirement of ECT2's m6A binding for functionality, implies that ALL of the Arabidopsis ECTs are capable of binding m6A transcripts – an important and highly significant finding.

We agree, and have now clarified this conclusion that was not put clearly enough forward in the originally submitted version.

6) The structural analysis of the disordered domains suggests that charge distribution and ability to form droplets may be playing an important role in determining ECT function as a te234 complimenter.

7) The improved phylogenetic tree is a useful addition to the field and now clearly shows the division of higher plant ECTs into the four sub groups.

Overall, this is a thorough and rigorous piece of work that begins to answer one of the key questions in the plant mRNA methylation field and opens new avenues for research. Open questions remain, particularly around the mRNA targets of ECT9 (and ECT1 and ECT11), and developmental effects such as trichome branching (strongly influenced by m6A level and the te234 mutation), but these will likely be addressed in follow up work.

We thank the reviewer for these comments. Indeed, we believe that our study does not provide all the answers, but rather clarifies fundamental basic principles that remained completely unknown, thereby setting the frame and direction for future work.

Reviewer #2: This manuscript reveals that "conservation and diversification of the molecular functions of YTHDF proteins" is mainly introduced that the m6A/YTHDF axis controls cellular proliferation and organ growth in plants, the same molecular functions that allow ECT2/3/4 to regulate stem cell proliferation. Lineage-specific neo-functionalization of ECT1, ECT9 and ECT11 happened after late duplication events. The expression pattern of ECT5/6/7/8/10 may be used in specific biological contexts response to stimuli, suggesting different molecular activities of YTHDF proteins. This manuscript needs improvements before it could be considered for publication.

First, the data in this manuscript are roughly descriptive. Detailed molecular mechanisms are still lacking. A possible way to improve is to show how the different YTHDF readers bind to m6A to determine cytoplasmatic properties of target transcripts.

In our opinion, an interesting paper worthy of publication in a journal for a broad audience such as *PLoS Genetics* has to offer some conceptual advance that is general enough in nature to appeal to researchers whose interests cover many different fields. One way to achieve such broad appeal is through the deciphering of detailed molecular mechanisms. It is not the only way, however. Sometimes very influential papers describe a new phenomenon without delving into mechanistic understanding at all. There are numerous examples of this even in the era of modern molecular biology: the realization that the *Drosophila melanogaster* Toll gene is required for innate immunity, the demonstration that organogenesis in plants initiates at auxin maxima, the observation that transgene expression can cause silencing of endogenous genes in Petunia, and the observation that several animal species produce endogenous small silencing RNAs, are all examples of purely descriptive papers with a substantial impact in their respective fields. We mention this neither to school the reviewer, nor to claim that our paper is comparable to these major contributions, but simply to support the argument that a focus on deciphering molecular mechanisms in detail is not a mandatory requirement for research on molecular biology to be of broad interest.

The present paper is an example of a study whose main objective is not to decipher a molecular mechanism in detail. It aims to elucidate whether the remarkable diversification of YTHDF proteins seen in most higher plants reflects a functional diversification such that the different proteins have genuinely different molecular functions, or whether the many proteins can perform largely the same function molecularly. A clear answer to this simple question is key to understand how the m^6A-YTHDF

axis is being employed to control different aspects of plant biology. As stated by Reviewer 1, our experimental approach gave clear results that allowed us to give a definitive answer to this basic question. In his words: '*This is a thorough and rigorous piece of work that begins to answer one of the key questions in the plant mRNA methylation field and opens new avenues for research'.*

In addition, our study does in fact deliver some mechanistic insight that we now highlight more clearly with a small, dedicated paragraph in the Discussion under the subheading '*Inferences on molecular properties of land plant YTHDF proteins*' (lines 633-655 of the revised version). For example, we clearly demonstrate that the intrinsically disordered regions of YTHDF proteins are major sites of functional diversification, but also that substitutions in the YTH domains play a role. These are important conclusions, as they establish for the first time an example of how functional diversification of YTHDF proteins can be achieved in any organism, and even point to some molecular properties that are likely to play a role in this diversification. In conclusion, while we agree with the reviewer that "detailed molecular mechanisms are still lacking" we cannot see why this would be an argument for not publishing the paper. It contains very important conclusions as it is (also supported by Reviewer 1's comments), and it adds some important mechanistic understanding to YTHDF function, even if many details are still not understood (as is also the case for the much more intensely studied vertebrate YTHDF proteins).

Finally, we are unsure what exactly is meant by **'***A possible way to improve is to show how the different YTHDF readers bind to m6A to determine cytoplasmatic properties of target transcripts***'.** How YTHDF proteins bind to m^{6}A and how they determine cytoplasmic properties of target transcripts are likely to be two different, if clearly linked, things. Binding m^{6}A is obviously a prerequisite for determining target transcript properties, but how YTHDF proteins do this, is a problem that has already been solved: the YTH domain contains a positively charged cleft that binds indiscriminately to single-stranded RNA and a hydrophobic pocket that accommodates the methylated adenosine to yield 10-20-fold higher affinity for methylated RNA than for unmethylated RNA. Extensive literature on that mechanism exists, mainly from 2014 and 2015, and it is cited in the Introduction.

Second, the presentation is suggested to be improved. Here are some specific suggestions:

1) Figure1 "Phylogenetic analysis of YTHDF proteins in land plants" should mark more YTHDF proteins information about Arabidopsis, the paper mainly uses Arabidopsis to carry out research.

We regret to say that it is not clear to us what the reviewer wants us to do here. We have marked all 11 arabidopsis YTHDF proteins in the phylogenetic tree, and their unique gene identifiers (AGIs), complete amino acid sequences marking the position of the YTH domain, protein sequence alignments, evolutionary distances, AlphaFold structure prediction, and IDR analyses are provided elsewhere in the manuscript and supplementary information. We do not see the need for adding any of these data on top of the phylogenetic tree in Fig. 1, and we cannot envision which additional information may be missing. However, we will gladly follow any clear suggestions that may improve the quality of the manuscript in that regard. Please note, however, that **Figure 1 has now been considerably extended and improved following the criticism of Reviewer 3**, as explained in the subsequent section.

2) The paper includes some unreported mutants, such as "ect1-1 (SAIL_319_A08), ect1-2 (GK-547H06), ect1-3 (SALK_059722), ect2-3, ect3-2, ect2-3/ect3-2 (Gde23), ect2-3/ect3-2/ect4-2 (Gte234) and ect2-1/ect3-1/ect4-2", the author can show some identification of mutants in supplemental data.

The mutant lines *ect2-3*, *ect3-2*, *Gde23* (*ect2-3/ect3-2*) and *te234* (*ect2-1/ect3-1/ect4-2*) were reported back in 2018 (Arribas-Hernández et al., Plant Cell), and *Gte234* (*ect2-3 ect3-2 ect4-2*) in 2020 (Arribas-Hernández et al., Development). This information is available in lines 549-553 at the beginning of the Methods section ('*Plant material and growth conditions*') of the former version of the manuscript: '*The mutants ect2-3, ect3-2, ect2-3/ect3-2 (Gde23), ect2-3/ect3-2/ect4-2 (Gte234) and ect2-1/ect3-1/ect4-2 (te234) used for genetic crosses or as background to produce transgenic lines, and the primers for genotyping the corresponding alleles, have been previously described (Arribas-Hernández et al, 2018; Arribas-Hernández et al, 2020)*'. **To make this information easier**

to find, we have added references to these publications in line 390 of the current Results section, and to the table in S14A Fig, while the detailed descriptions remain in Methods as they were in the first submission.

Regarding *ect1* mutant alleles, we are not completely sure of what the reviewer means by 'some identification', as qPCR showing absence of mRNA spanning the T-DNA insertions in *ECT1* is provided. Nevertheless, this comment made us consider that perhaps genotyping gels may be informative, and we hope that this is what the reviewer refers to. Hence, **we have included the genotyping results obtained for the identification of plants carrying homozygous T-DNA insertions in *ECT1* in the lines SAIL_319_A08 (*ect1-1*), GK-547H06 (*ect1-2*) and SALK_059722 (*ect1-3*), obtained from the Nottingham Arabidopsis Stock Center. This information is now in S13 Fig.**

3) In Fig. 3H, there is no difference significance analysis for the columns, the bar value of column is a little high, it is recommended to repeat this experiment.

We apologize for not having done statistical analysis of the qPCR data. We have now performed t-tests to compare the expression between wild type and each T-DNA line for each primer set, obtaining highly significant p-values for the above-mentioned regions regardless of the variance among samples. Hence, it is not necessary to repeat the experiment, but **we have added the results of the statistical test to Fig 3H and its corresponding legend**. **The supporting numerical data is also included as S9 Dataset.**

4) The font size of line 150 and line 151 is different, please use the same font size.

Indeed, a few lines had a different font size in the first submission. We apologize for this oversight, and acknowledge the reviewer for bringing it to our attention, as it has prompted us to **ensure a homogenous font size in the text of the revised version**. In connection to that, we would like to clarify that we have now embeded the captions of the main figures after their first mention within the text, as required for *PLoS Genetics*. To facilitate the distinction between captions and main text by the reviewers, we have formatted the captions with smaller case and line spacing, as there are no indications for the typography size of the captions in the submission guidelines.

5) The reference in line 685 of the article is incorrectly cited.

This was a formatting issue in Endnote that is now fixed. We thank the reviewer for noticing that problem.

6) The line 854 "Tesei G, Lindorff-Larsen K, 2022." should be changed to "Tesei G, Lindorff-Larsen K (2022)".

Because *PLoS Genetics* does not require specific formatting for first submissions, the initial referencing style was chosen according to our writing convenience, and it was one in which the publication years were fashioned between parentheses, that were indeed missing in the indicated reference. Nevertheless, we have now re-formatted the references with the *PLoS* style (*Vancouver*), for which the publication year should not be between parentheses. Additionally, because this particular bioRxiv article is now in Open Research Europe (2023), this reference is changed altogether. The final version, complying with *PLOS Genetics* reference formatting style, is now:

126. Tesei G, Lindorff-Larsen K. Improved predictions of phase behaviour of intrinsically disordered proteins by tuning the interaction range [version 2; peer review: 2 approved]. Open Research Europe. 2023;2(94). doi: 10.12688/openreseurope.14967.2.

7) In the middle of title of citing references should be a lowercase letter, such as the lines 644, 647,660…

Title case (all main words are capitalized, and most minor words are not) is the formatting style of choice for many journals, including the ones in the examples mentioned by the reviewer:

- *Cell Reports* (line 644, https://doi.org/10.1016/j.celrep.2018.10.020)
- *The Plant Cell* (line 647, (https://doi.org/10.1105/tpc.17.00833)
- *Biochemistry* (line 660, (https://pubs.acs.org/doi/10.1021/acs.biochem.8b01166).

On the other hand, **sentence case** (only the first word of the text, proper nouns, and genus names are capitalized) is the choice of journals like *Development* (https://doi.org/10.1242/dev.189134) and *PLoS Genetics* itself. Because in the submission guidelines we have only found instructions for the actual title of our article but not for the reference list, we have directly checked reference lists in other *PLoS Genetics* articles, finding that **both title and sentence case styles co-occur**, e.g. in the recent publication: '*Genomic conservation of crop wild relatives: A case study of citrus*' (https://doi.org/10.1371/journal.pgen.1010811):

18. Bett JS. Proteostasis regulation by the ubiquitin system. Essays Biochem. 2016;60(2):143–151. pmid:27744330

19. Marshall RS, Vierstra RD. Dynamic Regulation of the 26S Proteasome: From Synthesis to Degradation. Front Mol Biosci. 2019;6:40. pmid:31231659

Hence, respecting the style of the titles in the original journals seems to be the choice of *PLoS Genetics* for the reference list, and, therefore, we have not taken any action to change this specific aspect of reference formatting. Nevertheless, we will be glad to comply with any style change the editors may require.

8) Many formatting errors in this manuscript. It is suggested that this manuscript should carefully proofread.

We have carefully read the manuscript to identify any additional formatting issues, and performed the **five minor corrections** listed below. We thank the reviewer for making us aware that the formatting was not perfect in the first submitted version, and for pointing out some required improvements.

1) Capitalization of *ect4* in line 97, as it refers to the gene and not to a mutant allele.

2) Update of the name of the ribosomal protein *RPS5A/uS7B* to *US7Y* according to the latest proposed nomenclature (Scarpin *et al*, 2023).

3) Removal of italicization of '*ECT1/9/11'* in line 362, as it refers to the proteins and not to the genes.

3) In line 441 of the first submission, there was a 'be' that should not have been there, as it was incorrectly left after a modification in the grammar during editing. '… *a thorough inspection of the fluorescence intensity in all primary transformants revealed that the transgene was only expressed very weakly in a reduced subpopulation of plants, possibly be due to the tendency to trigger silencing …'*. This error is now amended.

4) A comma in line 449 that should be a full stop is now corrected: '*… these IDR characteristics correlate with the ability to fulfill ECT2/3/4 function in leaf formation (Fig. 6E-H), Hence, our results demonstrate that the core molecular activity of Ath ECT2/3/4 was present in the first land plants…*'.

5) In line 566, we have changed *Arabidopsis* (genus) for arabidopsis (currently accepted common name for *Arabidopsis thaliana*) for coherence.

Reviewer #3:

This study conducted phylogenetic analyses of YTHDF proteins, and performed a series of functional complementation assay, to investigate its molecular function in plants. The authors showed that YTHDF homolog was present in the early-diverging land plants and remained conserved over its diversification. The authors mainly focused on YTHDF protein paralogs in A. thaliana, and less efforts were paid on YTHDF protein homologs in other representative species in the main lineages of green plants. **The complement assays of YTHDF protein paralogs in A. thaliana is insufficient for the interpretation of function conservation or diversification**, and **it would be better to investigate the gene expression and phenotypes in corresponding mutants.**

The reason why the reviewer qualifies our functional complementation assay as insufficient is unclear, as this statement is not accompanied by any solid argumentation, or indeed any argument at all. Our study gives clear, robust answers to whether or not the genes in question encode proteins with the molecular functions of ECT2/3/4 needed for leaf formation. The clearly different activity of 3

(ECT1/9/11) out of the 11 arabidopsis YTHDFs is sufficient to conclude that **diversification** of the molecular function does exist among plant YTHDF proteins (Reviewer 1: '*The lack of complementation by ECT9, ECT1 and ECT11 is curious. The elegant domain swap experiments indicate that this is likely due to neofunctionalisation of the disordered domains*'), and this conclusion is further supported by the in-depth genetic study of one of the three proteins (Reviewer 1: *'The finding that ECT1 does not rescue ECT2/3/4 is unexpected and appears to be primarily due to altered functionality of the disordered N terminus'*). On the other hand, the fact that proteins from clades that diverged prior to angiosperm diversification can perform the same molecular functions is, in itself, sufficient to conclude that there is also a high degree of **conservation** (Reviewer 1: *'The complementation by at least some members of each of the 4 ECT sub groups suggests that the primordial function has been retained'*). Finally, the fact that a protein from an organism that diverged from the rest of the land plant lineage more than 400 million years ago, can fulfill the same molecular function, is solid proof of **conservation** (Reviewer 1: '*The fact that the single YTHDF found in liverworts can rescue the ECT2/3/4 phenotype is a critical finding as it indicates a conserved and ancient function for basal plant YTHDF function*').

In summary, given that the reviewer does not explain the reasons to claim that our assay is insufficient to draw conclusions on functional conservation/diversification, and that this concern is not shared by any other reviewer, we ask the editor to please disregard this point of criticism.

We also note that the statement '*it would be better to investigate the gene expression and phenotypes in the corresponding mutants*' actually strongly supports the importance of the results reported here. Thinking further, one would have to ask oneself: "Exactly which mutants should I combine?" With 8 YTHDF proteins of completely unknown function, and considering that the genetic interaction between these and the 3 already studied must also be addressed, the number of possible mutant combinations, with 11 genes, is quite baffling. With the results reported here, a framework is built to further study biological function of plant YTHDF proteins, because different functional groups are now clearly defined.

The actions taken regarding the comment '*less efforts were paid on YTHDF protein homologs in other representative species in the main lineages of green plants*' are described in response to the more specific requests from the reviewer on that matter below.

There are some concerns that needed to be clarified.

1.The results section should be carefully checked and rephrased:

(1) combine some results and reduce the use of subheadings;

We consider that a style using short results sections preceded by subheadings improves clarity, makes the information easier to digest, and facilitates browsing for specific information throughout the text. In that regard, the Reviewer 1 says: '*The manuscript is very well written, the experiments are clear and easy to follow*', and Reviewer 2 does not have any objections on the subject. Hence, because this is largely a matter of personal taste and the suggestion is not supported by any other reviewer, we leave the subheadings and sections as they are.

(2) reduce the description of previous results, for example, Line 116-125;

Lines 116-125 do not merely describe previous results, but rather explain why it is relevant and necessary to build a phylogenetic tree that includes clades intermediate between bryophytes and angiosperms, despite the fact that a detailed phylogenetic tree has already been published (Scutenaire et al., 2018). Thereby, it situates our analysis within the current state-of-the-art. Furthermore, the result referred to by the reviewer is a phylogenetic analysis rich in angiosperm species that is used throughout the text regarding late evolutionary events of ECTs (e.g. in lines 421 and 505). Therefore, a description of that previous analysis is necessary. Finally, definitions of plant taxa such as *liverworts*, *bryophytes* or *angiosperms* are helpful for readers unfamiliar with botanic terms. In summary, we consider that we cannot reduce much the content of these lines without compromising clarity, but **we have shortened the text by removing one sentence that was not strictly necessary**.

We have scanned the results section with that comment in mind, to find other descriptions of previous results. In almost all cases, they are brief accounts of the information that is needed to understand our experiments, or to interpret our results within specific contexts. We believe that moving such short descriptions to the introduction would weaken the connection with the relevant experiments or results. Because this objection is not raised by other reviewers, and Reviewer 1 notes that the manuscript is easy to follow, we have found it wise to leave most of these brief sentences as they are. The reasons are discussed in a case-to-case manner below. Nevertheless, **we have moved the description of ECT2-condensates to the Introduction following the reviewer's suggestion, as we agree that this is a more lengthy description of previous results, and therefore it is better placed in the Introduction.**

The other cases of description of previous results found throughout the results section are:

- The sentence in lines 204-206 '*This conclusion is not trivial, as some eukaryotes lacking canonical m^{6}A-methyltransferases encode YTH domain proteins (Balacco & Soller, 2019) and, indeed, the YTH domain of fission yeast Mmi1 does not bind to m^{6}A (Wang et al, 2016)*' is necessary to explain why the m^{6}A-binding capacity of the yet-unstudied ECTs could not just be inferred by the presence of a YTH domain, highlighting the importance of the result. This view is shared by Reviewer 1: *'… implies that ALL of the Arabidopsis ECTs are capable of binding m6A transcripts – an important and highly significant finding'.*

- Lines 229-231 '*we tested whether the same results could be reproduced in plants lacking RNA-DEPENDENT RNA POLYMERASE 6 (RDR6), a gene essential for the biogenesis of transgene-derived short interfering RNAs (siRNAs) (Dalmay et al, 2000; Mourrain et al, 2000)*' are indispensable for readers not familiar with the RNAi pathway in plants to understand the use of the *rdr6* mutant in our study. Similarly, the short description of *rdr6* single mutants in lines 234-235 '*we constructed rdr6-12/te234 mutants that exhibited both the narrow, downward-curling leaf phenotype of rdr6 mutants (Peragine et al, 2004)*' is required to validate and interpret the phenotype of the quadruple *te234/rdr6-12* mutant constructed by us.

- In lines 279-282 ('*ECT1 promoter-reporter fusions (Ok et al, 2005) and mRNA-seq data (Zhang et al, 2020) suggest that expression levels and tissue specificity of ECT1 are comparable to that of ECT4, whose activity is easily revealed as an enhancer mutant of ect2/ect3 (Arribas-Hernández et al., 2018)*'), the brief description of previous results on ECT1 and ECT4 is necessary to argument why ECT1 is a good candidate for validation of our assay.

- The brief reference to the previously published expression patterns of ECT2/3/4 (lines 287-291, *'The pattern of turquoise fluorescence was strongly reminiscent of that of fluorescent ECT2/3/4 fusions (Arribas-Hernández et al., 2018)'*) is necessary for the direct comparison of these results with that of ECT1-FLAG-TFP. Accordingly, the description of the new and old results is combined in the same sentence.

- Finally, lines 369-374 described previous results supporting phase-separation properties of ECT2 and human YTHDFs and, as mentioned above, **we have moved this information to the Introduction following the reviewer's recommendation.** We acknowledge the reviewer for prompting us to perform this improvement,

We have not found other instances of mentions to previously published results within the Results section apart from these.

(3) check the result and move the descriptions of method to the method section, for example, Line 162-170.

Lines 162-166 do not describe a method, but rather justify the choice of promoter for the complementation assays. That is not at all a technical detail such as e.g. the choice of cloning strategy would be. The promoter determines the expression domain, and therefore it has fundamental biological implications that need to be put forward. The rest of the paragraph (lines 166-170) describes <u>results</u> of experiments conducted in this manuscript.

Browsing through the results section, we have not found any instances of methodological descriptions. The only content similar to that are the accounts of the biological, evolutionary or

molecular basis for the choice of i) plant species for phylogenetic studies, ii) genetic backgrounds, scoring/quantification/thresholding strategy for the complementation assays, and iii) protein regions for structural vs. biophysical analyses and for the design of chimeric constructs. In all cases, the explanation is necessary for understanding the experimental approach. On the other hand, the methodological details for the experiments and analyses are abundantly provided in the methods section, as they must be.

2. The manuscript aimed to investigate the molecular function of plant YTHDF proteins. However, **the taxa samplings of plant genomic data appear to be deficiency, only containing few land plants** and ignoring the early-branching green plants (such as chlorophytes and charophytes).

There are pros and cons in using large numbers of species for phylogenetic analyses, and the optimal number depends on the objective of the analysis. While using many species certainly increases the accuracy of the inferred phylogenetic relationships, a clear disadvantage is the reduced overview and clarity, as the typography needs to shrink considerably to fit the many proteins, and the amount of information can become overwhelming for non-taxonomists. This problem is exacerbated for protein families with large number of paralogs.

Because the main focus of this article is not the detailed determination of the phylogeny of Viridiplantae YTHDF proteins, as it is rather a functional study of arabidopsis YTHDFs within the context of land plant YTHDF evolution, we chose to prime clarity over detail in our first submission. In that regard, Reviewer 1 seems to appreciate the analysis done with 10 well-selected species representative of all main taxa on land plants, as he says: '*The improved phylogenetic tree is a useful addition to the field and now clearly shows the division of higher plant ECTs into the four sub groups'.* Nevertheless, we believe that we have now found a solution to improve accuracy without compromising clarity. In order to satisfy the requirement of Reviewer 3 for a larger number of species, **we have extended our analysis to 32 taxa of land plants. We have also inferred the relationship between these and the orthologs from 8 species of green algae, and rooted the tree with 3 taxa outside but closely related to Viridiplantae**. To balance the trade-off between the increased detail and the former clarity, we have provided a simplified version of the analysis in the main figure (Fig 1B), labeling only the proteins from key taxa and the most important nodes, and presented the full-labeled version with all proteins and aLRT scores of all nodes in supplementary (S1 Fig), where there is no requisite for minimum typography size. Of note, **the result is almost identical to the one in the first submission** in terms of overall tree topography, clade definition and assignment to clades of the proteins present in both analyses. Furthermore, **the conclusions relevant to our study have not changed**. We hope that this number of species fully satisfies the reviewer's request, as it is roughly the same number of land plant species as in the phylogenetic tree published in Plant Cell (Scutenaire et al., 2018). The most important difference between the two analyses is that Scutenaire et al. focused on angiosperms (30 species of angiosperms and 3 of bryophytes), while we have spread the analysis among all main taxa, with 5 bryophytes, 3 lycophytes, 6 ferns, 2 gymnosperms, 2 basal angiosperms, 2 magnoliids, 4 monocots and 8 dicots, in addition to 4 chlorophytes and 4 streptophyte algae (charophytes), and 3 outgroup species. The only bias in the sampling is inevitably caused by the great differences in the number of species whose genomes have been sequenced among taxa, and the fact that few algae encode YTHDF proteins.

Regarding the reviewer comments on '*ignoring early-branching green plants when the study aims to investigate molecular function of plant YTHDF proteins'*, we would like to remark that our study aims to investigate the molecular function of YTHDF proteins in **land plants** (embryophytes), and that is why we have not mentioned green algae such as chlorophytes and charophytes. In that respect, we apologize for the loose use of the term 'plants' throughout the text. Indeed, '*Plantae sensu strictissimo*' refers only to land plants ('*All green plants above green algae*' (Margulis, 1971)), '*Plantae sensu stricto*' are Viridiplantae (green plants) comprising streptophytes (inc. land plants) and chlorophytes, while '*Plantae sensu lato*' includes red algae (rhodophytes) and glaucophyte algae in addition to green plants—all organisms whose common ancestor acquired primary chloroplasts directly by engulfing cyanobacteria—, that is, Archaeoplastida (Cavalier-Smith, 1981). Hence, the inaccurate term 'plants' is now replaced by the more specific 'land plants/embryophytes', 'Viridiplantae' or 'Archaeoplastida' according to its exact meaning in each instance. Nevertheless,

because we agree that the phylogenetic tree of land plant YTHDFs would benefit from a solid root, we have now included green algae in the phylogenetic analysis (Figs 1 and S1-S4), complying with the reviewer's request.

3. For the phylogenetic analyses, **why did the authors trimmed from their N-termini to discard intrinsically disordered regions with poor sequence conservation. In general, the protein sequences of homologs should be aligned, and then the multiple sequence alignments should be trimmed with proper threshold values.**

The phylogenetic analysis in the first submission was done following the procedure used by (Scutenaire *et al.*, 2018), as they used the sequences of the YTH domains extended by adding the flanking regions that align to the structural elements outside the canonical YTH in human YTHDF1 (according to the structure by (Xu *et al*, 2015)). Scutenaire's article in Plant Cell is, to our knowledge, the best phylogenetic study on YTH domain proteins of higher plants. Nevertheless, **we have followed the reviewer's recommendation for the new tree, as using a threshold of conservation/quality values to trim the alignment retains roughly the same regions as before**. As expected, the resulting tree is almost identical in terms of overall topography, clade definition and assignment of the proteins present in both analyses to clades. Furthermore, both trees largely agree with that of Scutenaire *et al.* (2018). **The detailed account of the trimming procedure is now documented in the Methods section, in S2-S3 Datasets, and in S23 Fig.**

4. For the complement assays, the authors mainly focused on YTHDF protein paralogs in A. thaliana, and **few evidence were shown to elucidate the protein evolution in plants. It would be great interests to determine the molecular functions of YTHDF protein homologs in several representative species in the main lineages of green plants, including chlorophytes, charophytes, bryophytes, ferns, gymnosperms.**

Regarding the absence of attempts to elucidate the molecular functions of chlorophyte and charophyte YTHDF proteins, we have explained above that this study is restricted to **land plants**. That is already a vast group of organisms, in particular when compared to studies commonly restricted to one species (*S. cerevisiae,* or *D. melanogaster,* or *C. elegans,* or *E. coli,* or *A. thaliana*, etc.), small groups such as mammals (typically mouse and human), or e.g. a few angiosperms. Indeed, none of the other two reviewers has considered that including green algae in the study is necessary for neither the validity of the conclusions nor for the interest of the article. In fact, it is unclear why Reviewer 3 draws the line on Viridiplantae and not, for example, restricts it to Streptophyta (excluding Chlorophyta), or expands it to Archaeoplastida (including Rhodophyta and Glaucophyta), or even further down the tree of life. Such limits seem to be rather arbitrary, and we believe that the monophyletic clade of Embryophyta (land plants) is a clearly defined group with a distinct ecological relevance, unmeasurable economic value, and huge scientific interest. However, during the exercise of finding algal YTHDFs to aid rooting the tree of land plant YTHDFs as suggested by this reviewer, we noticed that chlorophytes seem to have lost YTHDF proteins with the exception of a few genera from Mamiellophyceae, an early-diverging class. Furthermore, all chlorophyte and some charophyte YTHDFs are highly divergent, and some even include additional domains. Hence, it is entirely possible that the function of YTHDF proteins in green algae have diverged considerably from the last common ancestor of the Viridiplantae linage, further indicating that these proteins should be subject of independent dedicated studies. Because these observations are indeed interesting, but their consideration in the results section would detract from the main focus of our study, **we have illustrated these findings as supplementary figures (S2-S4 and S22), and a discussion on the meaning of the presence or absence of YTHDF proteins in green, red and glaucophyte algae, and their peculiarities, can now be found in a dedicated section of the Discussion.** We expect that this preliminary overview and discussion may encourage dedicated studies from groups working with these types of organisms.

Regarding the other three groups requested by the reviewer (bryophytes, ferns and gymnosperms), liverworts such as *Marchantia polymorpha* (*Mpo*) are currently considered bryophytes (Harris *et al*, 2020), and we have determined that *Mpo* DFE can fulfill the molecular functions of *ECT2/3/4* in arabidopsis. **Because the last common ancestor of bryophytes and angiosperms is also ancestor of ferns and gymnosperms, the functionality of *Mpo* DFE in arabidopsis indicates the existence of a conserved molecular mechanism in the last common ancestor of**

bryophytes, ferns, gymnosperms and angiosperms. Quoting Reviewer 1, '*The fact that the single YTHDF found in liverworts can rescue the ECT2/3/4 phenotype is a critical finding as it indicates a conserved and ancient function for basal plant YTHDF function*'. Therefore, the interest of testing the many YTHDF paralogs in ferns and gymnosperms is restricted to determine whether some of them have lost that common function, similarly to ECT1/9/11 in arabidopsis. **Although such studies may be important to understand fern and gymnosperm molecular biology, they are not necessary for the conclusion of a conserved molecular mechanism in all land plants. Nevertheless, to satisfy the reviewer's specific interest in ferns and gymnosperms, and to extend the conclusions of the analysis of the IDRs to the main taxa of land plants**, **we have expanded our analysis of biophysical properties of IDRs (charge distribution and simulation of phase-separation propensity) to YTHDF proteins from mosses, lycophytes, ferns and gymnosperms (S21 Fig)**. Gratifyingly, these analyses demonstrate that all these organisms encode at least one YTHDF protein whose IDR has the combination of biophysical properties seen in *Mpo* DFE and ECT2/3/4/5/6/7/8/10, while those with many YTHDFs also contain a few paralogs whose IDRs diverge in ways similar to what we describe for ECT1/9/11.

5. This section, "The ability to complement ect2/3/4 does not simply follow affiliation with phylogenetic clades", is of interests, **but lack extensive discussions about this pattern**.

The implication of this finding is extensively discussed in lines 423-426 of the first submitted version '*These results suggest that the common ancestor of YTHDF proteins in land plants may have had a molecular function related to that of the modern arabidopsis ECT2/3/4, and that this function has been conserved in the least-divergent members of the DF-A/B/C/D clades'* and followed up by the *Marchantia polymorpha* DFE complementation experiment (*'To test whether the common ancestor of plant YTHDF proteins indeed had a molecular function similar to that of the modern Arabidopsis ECT2/3/4, we subjected Mpo YTHDF (Fig. 2A) to our functional assay, as bryophytes are the earliest-diverging taxon of land plants*'. Finally, the last paragraph of the article discusses the possible biological significance of this result.

Have all data underlying the figures and results presented in the manuscript been provided?

Large-scale datasets should be made available via a public repository as described in the PLOS Genetics data availability policy, and numerical data that underlies graphs or summary statistics should be provided in spreadsheet form as supporting information.

Reviewer #1: Yes

Reviewer #2: None

Reviewer #3: Yes

The answer 'none' from Reviewer 2 is neither correct nor fair. In the first submission, we already provided as supplementary information: i) the sequences of all oligonucleotides used for the study (primer sets for genotyping, qPCR, probe production and cloning), ii) the isoform for each YTHDF-mCherry construct, iii) the amino acid sequences of all studied proteins, specifying the different regions used for each analysis, iv) all protein sequence alignments, v) the computed phylogenetic tree in Newick format, vi) the data matrix of evolutionary distances, vii) the detailed account, on the sequence alignment and protein structure, of the substitutions in conserved residues of YTH domains of ECT1/9/11 summarized in Fig 5A, viii) the prediction of disordered regions and SLIMs in *Ath* ECT IDRs, ix) the raw counts of complementation of primary transformants in a graphic format together with representative images of T1 seedlings (bright field and mCherry fluorescence) for all genotypes tested, and x) the transformation efficiency of every single transformation accounted for. Additionally, we included photos of additional developmental stages and controls, the detailed measurement of possible developmental phenotypes of *ect1*-mutant combinations, movies of the phase transition simulations, and more. In other words, we have put an enormous effort in offering neat supplementary figures (15 in the first submission and now 23 to accommodate the reviewers' requests) with abundant additional information, not only to maximize transparency and reproducibility, but also to provide all the data that we have gathered in a well-organized and easy-to-use manner. The purpose is that other groups can benefit from the time we have spent collecting,

analyzing, organizing and presenting the data for further studies. In addition, **we have now added the raw counts described in (ix) and the data for the qPCR analysis on *ECT1* alleles (Fig 3H) in spreadsheet form (S6 and S9 Datasets respectively). Finally, the large amount of data and analysis scripts of IDR simulations have been placed in a Zenodo repository (https://doi.org/10.5281/zenodo.8269719).**

We believe that uncropped images of gels and photographs of plants are sometimes required by some journals prior to publication, but we are uncertain on whether PLoS Genetics adheres to that policy or not. Because collecting and labeling such material in an organized manner is tedious and perhaps unnecessary, and we have not found instructions for it at the current stage, we will hold the preparation of such material for now, but we will gladly provide it if requested by the journal.

References

Arribas-Hernández L, Bressendorff S, Hansen MH, Poulsen C, Erdmann S, Brodersen P (2018) An m6A-YTH Module Controls Developmental Timing and Morphogenesis in Arabidopsis. *Plant Cell* 30: 952-967

Arribas-Hernández L, Simonini S, Hansen MH, Paredes EB, Bressendorff S, Dong Y, Østergaard L, Brodersen P (2020) Recurrent requirement for the m6A-ECT2/ECT3/ECT4 axis in the control of cell proliferation during plant organogenesis. *Development* 147: dev189134

Balacco DL, Soller M (2019) The m6A Writer: Rise of a Machine for Growing Tasks. *Biochemistry* 58: 363-378

Cavalier-Smith T (1981) Eukaryote kingdoms: Seven or nine? *Biosystems* 14: 461-481

Dalmay T, Hamilton A, Rudd S, Angell S, Baulcombe DC (2000) An RNA-dependent RNA polymerase gene in Arabidopsis is required for posttranscriptional gene silencing mediated by a transgene but not by a virus. *Cell* 101: 543-553

Harris BJ, Harrison CJ, Hetherington AM, Williams TA (2020) Phylogenomic Evidence for the Monophyly of Bryophytes and the Reductive Evolution of Stomata. *Current Biology* 30: 2001-2012.e2002

Margulis L (1971) Whittaker's five kingdoms of organisms: minor revisions suggested by considerations of the origin of mitosis. *Evolution* 25: 242-245

Mourrain P, Beclin C, Elmayan T, Feuerbach F, Godon C, Morel J-B, Jouette D, Lacombe A-M, Nikic S, Picault N *et al* (2000) *Arabidopsis SGS2* and *SGS3* genes are required for posttranscriptional gene silencing and natural virus resistance. *Cell* 101: 533-542

Ok SH, Jeong HJ, Bae JM, Shin JS, Luan S, Kim KN (2005) Novel CIPK1-associated proteins in Arabidopsis contain an evolutionarily conserved C-terminal region that mediates nuclear localization. *Plant Physiol* 139: 138-150

Peragine A, Yoshikawa M, Wu G, Albrecht HL, Poethig RS (2004) SGS3 and SGS2/SDE1/RDR6 are required for juvenile development and the production of trans-acting siRNAs in Arabidopsis. *Genes Dev* 18: 2368-2379

Scarpin MR, Busche M, Martinez RE, Harper LC, Reiser L, Szakonyi D, Merchante C, Lan T, Xiong W, Mo B *et al* (2023) An updated nomenclature for plant ribosomal protein genes. *Plant Cell* 35: 640-643

Scutenaire J, Deragon J-M, Jean V, Benhamed M, Raynaud C, Favory J-J, Merret R, Bousquet-Antonelli C (2018) The YTH Domain Protein ECT2 Is an m6A Reader Required for Normal Trichome Branching in Arabidopsis. *Plant Cell* 30: 986-1005

Wang C, Zhu Y, Bao H, Jiang Y, Xu C, Wu J, Shi Y (2016) A novel RNA-binding mode of the YTH domain reveals the mechanism for recognition of determinant of selective removal by Mmi1. *Nucleic Acids Res* 44: 969-982

Xu C, Liu K, Ahmed H, Loppnau P, Schapira M, Min J (2015) Structural Basis for the Discriminative Recognition of N6-Methyladenosine RNA by the Human YT521-B Homology Domain Family of Proteins. *Journal of Biological Chemistry* 290: 24902-24913

Zhang H, Zhang F, Yu Y, Feng L, Jia J, Liu B, Li B, Guo H, Zhai J (2020) A Comprehensive Online Database for Exploring ~20,000 Public Arabidopsis RNA-Seq Libraries. *Molecular Plant* 13: 1231-1233
