# Supplementary material for: Insights into the conservation and diversification of the molecular functions of YTHDF proteins
Source: PLoS Genet. 2023 Oct 10;19(10):e1010980. doi: 10.1371/journal.pgen.1010980 (PMC10617740; doi:10.1371/journal.pgen.1010980)
Supplement: Attachment — Submitted filename: Review.docx [file pgen.1010980.s039.docx]

Adenosine methylation in mRNA is known to play important roles in plant development, and the YTH (plant ECT) family of proteins are known to be the main “readers” that bind to RNAs containing m6A. However, much confusion and lack of information exists around how the binding of such proteins brings about normal growth. The eukaryote YTH family can be further divided into nuclear YTHDC-type and cytoplasmic YTHDF-type readers. In both plants and animals, it is the YTHDF-type that have been evidenced as being most critical for bringing about the majority of the m6A-dependent post transcriptional outcomes. In humans there are three YTHDFs, and roles in promoting mRNA translation, degradation or stabilization have variously been claimed, though there is a growing consensus that all promote destabilisation. Higher land plants have an expanded family of YTHDFs, and determining whether these all carry out the same function or have different (perhaps even opposing) functions is a pressing question in the field, which this paper seeks to address.

Overall, the manuscript is very well written, the experiments are clear and easy to follow and there are several important findings that will be of considerable interest to the field. Most notably;

1) The fact that the single YTHDF found in liverworts can rescue the ECT2/3/4 phenotype is a critical finding as it indicates a conserved and ancient function for basal plant YTHDF function.
2) The finding that ECT1 does not rescue ECT2/3/4 is unexpected and appears to be primarily due to altered functionality of the disordered N terminus. This might have been due to more complex regulation of ECT1, such as requirement for post translational modification. However, this is argued against by the lack of additional growth defect in the quadruple mutant.
3) The complementation by at least some members of each of the 4 ECT sub groups suggests that the primordial function has been retained. This suggests that the reason for expansion of this family in plants may reflect differences in expression/regulation of the different ECTs rather than evolution of different functions upon mRNA binding. However, the lack of complementation by ECT9, ECT1 and ECT11 is curious. The elegant domain swap experiments indicate that this is likely due to neofunctionalisation of the disordered domains.
4) The enhancement of the triple mutant phenotype following expression of ECT9 is particularly striking, especially as this seems to be dependent upon the presence of the existing te234 mutations. This may suggest an antagonistic function in this case and this is likely to become an active area of research.

The human YTHDF2 also enhanced the te234 phenotype - likely due to competition for m6A binding, but remains an interesting and potentially useful observation.

5) The combination of complementation experiments, domain swap-complementation and (in the case of ECT9) inhibition, taken together with the known requirement of ECT2’s m6A binding for functionality, implies that ALL of the Arabidopsis ECTs are capable of minding m6A transcripts – an important and highly significant finding.
6) The structural analysis of the disordered domains suggests that charge distribution and ability to form droplets may be playing an important role in determining ECT function as a te234 complimenter.
7) The improved phylogenetic tree is a useful addition to the field and now clearly shows the division of higher plant ECTs into the four sub groups.

Overall, this is a thorough and rigorous piece of work that begins to answer one of the key questions in the plant mRNA methylation field and opens new avenues for research. Open questions remain, particularly around the mRNA targets of ECT9 (and ECT1 and ECT11), and developmental effects such as trichome branching (strongly influenced by m6A level and the te234 mutation), but these will likely be addressed in follow up work.
